# Transmission of Multidrug-Resistant and Drug-Susceptible Tuberculosis within Households: A Prospective Cohort Study

**DOI:** 10.1371/journal.pmed.1001843

**Published:** 2015-06-23

**Authors:** Louis Grandjean, Robert H. Gilman, Laura Martin, Esther Soto, Beatriz Castro, Sonia Lopez, Jorge Coronel, Edith Castillo, Valentina Alarcon, Virginia Lopez, Angela San Miguel, Neyda Quispe, Luis Asencios, Christopher Dye, David A. J. Moore

**Affiliations:** 1 Wellcome Centre for Clinical Tropical Medicine, Imperial College London, London, United Kingdom; 2 Universidad Peruana Cayetano Heredia, Lima, Peru; 3 TB Centre, London School of Hygiene & Tropical Medicine, London, United Kingdom; 4 Johns Hopkins Bloomberg School of Public Health, Baltimore, Maryland, United States of America; 5 Laboratorio de Mycobacteriologia, Dirección Regional de Salud–Región Callao, Lima, Peru; 6 Unidad Técnica de TB-MDR, Ministerio de Salud, Lima, Peru; 7 Estrategia Sanitaria Nacional de Prevención y Control de la Tuberculosis and Laboratorio de Mycobacteriologia, Dirección de Salud II–Lima Sur, Lima, Peru; 8 Instituto Nacional de Salud, Lima, Peru; 9 Office for HIV/AIDS, Malaria, Tuberculosis and Neglected Tropical Diseases, World Health Organization, Geneva, Switzerland; University of Amsterdam, NETHERLANDS

## Abstract

**Background:**

The “fitness” of an infectious pathogen is defined as the ability of the pathogen to survive, reproduce, be transmitted, and cause disease. The fitness of multidrug-resistant tuberculosis (MDRTB) relative to drug-susceptible tuberculosis is cited as one of the most important determinants of MDRTB spread and epidemic size. To estimate the relative fitness of drug-resistant tuberculosis cases, we compared the incidence of tuberculosis disease among the household contacts of MDRTB index patients to that among the contacts of drug-susceptible index patients.

**Methods and Findings:**

This 3-y (2010–2013) prospective cohort household follow-up study in South Lima and Callao, Peru, measured the incidence of tuberculosis disease among 1,055 household contacts of 213 MDRTB index cases and 2,362 household contacts of 487 drug-susceptible index cases.

A total of 35/1,055 (3.3%) household contacts of 213 MDRTB index cases developed tuberculosis disease, while 114/2,362 (4.8%) household contacts of 487 drug-susceptible index patients developed tuberculosis disease. The total follow-up time for drug-susceptible tuberculosis contacts was 2,620 person-years, while the total follow-up time for MDRTB contacts was 1,425 person-years. Using multivariate Cox regression to adjust for confounding variables including contact HIV status, contact age, socio-economic status, and index case sputum smear grade, the hazard ratio for tuberculosis disease among MDRTB household contacts was found to be half that for drug-susceptible contacts (hazard ratio 0.56, 95% CI 0.34–0.90, *p* = 0.017). The inference of transmission in this study was limited by the lack of genotyping data for household contacts. Capturing incident disease only among household contacts may also limit the extrapolation of these findings to the community setting.

**Conclusions:**

The low relative fitness of MDRTB estimated by this study improves the chances of controlling drug-resistant tuberculosis. However, fitter multidrug-resistant strains that emerge over time may make this increasingly difficult.

## Abstract 2

In this prospective cohort study, Louis Grandjean and colleagues examine the relative fitness of multidrug-resistant versus drug-susceptible tuberculosis for transmission among household contacts in South Lima and Callao, Peru.

## Editors' Summary

**Background:**

Tuberculosis—a contagious bacterial disease that usually infects the lungs—is a global public health problem. Every year, 8.6 million people develop active tuberculosis (tuberculosis disease), and at least 1.3 million people die as a result, mainly in resource-limited countries. *Mycobacterium tuberculosis*, the bacterium that causes tuberculosis, is spread in airborne droplets when people with tuberculosis disease cough or sneeze. Consequently, an individual’s risk of contracting tuberculosis increases with his/her frequency of contact with people who have the disease; people who live in the same household as someone with tuberculosis disease are at particularly high risk. Other risk factors for contracting tuberculosis include living in crowded or insanitary conditions and being immunocompromised because of, for example, infection with HIV. The characteristic symptoms of tuberculosis disease are persistent cough, fever, weight loss, and night sweats. Diagnostic tests for the disease include sputum smear microscopy (microscopic analysis of mucus coughed up from the lungs), the growth of *M*. *tuberculosis* from sputum samples, and chest X-rays.

**Why Was This Study Done?:**

Taking several antibiotics (including rifampicin and isoniazid) daily for six months can cure tuberculosis, but the emergence of multidrug-resistant tuberculosis (MDRTB) is making the disease increasingly hard to treat. How badly MDRTB will affect tuberculosis control efforts is likely to depend on the relative “fitness” of multi-drug resistant and drug-susceptible *M*. *tuberculosis* strains. The fitness of a pathogen (infectious organism) is its ability to survive, reproduce, be transmitted, and cause disease in another host. Animal and laboratory studies indicate that drug-resistant *M*. *tuberculosis* strains are less fit than drug-susceptible strains, but these studies do not account for the clinical, environmental, and socio-economic variables that influence a patient’s ability to cause tuberculosis disease in a contact, and may not accurately measure the relative fitness of *M*. *tuberculosis* strains. In this prospective cohort study, the researchers estimate the fitness of drug-resistant tuberculosis relative to drug-susceptible tuberculosis by comparing the incidence of additional cases of tuberculosis disease in households with an MDRTB index case and the incidence in households with a drug-susceptible tuberculosis index case. A prospective cohort study follows a group of people over time to see whether specific baseline characteristics are associated with specific outcomes. The incidence of a disease is the number of new cases in a population over a given time period.

**What Did the Researchers Do and Find?:**

The researchers enrolled 1,055 household contacts of 213 MDRTB index cases (individuals whose disease was resistant to at least rifampicin and isoniazid) and 2,362 household contacts of 487 drug-susceptible tuberculosis index cases living in South Lima and Callao, Peru. During three years of follow-up, 35 (3.3%) of the household contacts of the MDRTB index cases and 114 (4.8%) of the household contacts of the drug-susceptible tuberculosis index cases developed tuberculosis disease. After adjusting for factors likely to affect the transmission of tuberculosis, such as HIV status, socio-economic status, and sputum smear grade of the index case (higher smear grades are associated with a higher risk of tuberculosis transmission), the hazard ratio for tuberculosis disease for household contacts of MDRTB index cases was half that of the household contacts of drug-susceptible tuberculosis index cases. That is, the household contacts of MDRTB index cases contracted tuberculosis disease half as often as those of drug-susceptible tuberculosis index cases.

**What Do These Findings Mean?:**

These findings indicate that, within households, MDRTB has a relatively low fitness compared to drug-susceptible tuberculosis. That is, at least during the first three years following exposure, individuals with MDRTB are less likely to transmit disease to their household contacts than individuals with drug-susceptible tuberculosis. These findings agree with those of previous animal and laboratory studies and with the findings of molecular epidemiology studies that have used genetic methods to estimate *M*. *tuberculosis* fitness within populations. Because the researchers did not genetically compare *M*. *tuberculosis* strains isolated from the index cases with strains isolated from the household contacts who developed tuberculosis disease, some of these contacts may have become infected outside the household. Moreover, it may not be possible to extrapolate these findings to the community setting. Nevertheless, the low relative fitness of MDRTB reported here improves our chances of controlling the spread of drug-resistant tuberculosis, with the proviso that the emergence of fitter MDRTB strains over time might yet threaten global tuberculosis control efforts.

**Additional Information:**

This list of resources contains links that can be accessed when viewing the PDF on a device or via the online version of the article at http://dx.doi.org/10.1371/journal.pmed.1001843.

- The World Health Organization provides information (in several languages) on tuberculosis and on multidrug-resistant tuberculosis; the *Global Tuberculosis Report 2014* provides information about tuberculosis around the world; a supplement to the report entitled *Drug-Resistant TB—Surveillance and Response* is available
- The Stop TB Partnership is working towards tuberculosis elimination and provides personal stories about tuberculosis (in English and Spanish); the Tuberculosis Vaccine Initiative (a not-for-profit organization) also provides personal stories about tuberculosis
- The US Centers for Disease Control and Prevention provides information about tuberculosis and about drug-resistant tuberculosis (in English and Spanish)
- The US National Institute of Allergy and Infectious Diseases also has detailed information on all aspects of tuberculosis
- MedlinePlus has links to further information about tuberculosis (in English and Spanish)

## Introduction

Natural selection of an infectious pathogen occurs as a consequence of differential reproductive success at the level of the gene or the organism during its interaction with the environment. The “fitness” of *Mycobacterium tuberculosis* is defined as the ability of the organism to survive in the host, reproduce, be transmitted, and cause disease in another host [1,2]. Mathematical models suggest that the scale of the future threat of multidrug resistance to tuberculosis control depends on both the relative and absolute “fitness” of multidrug-resistant and drug-susceptible *M*. *tuberculosis* organisms [3–5].

Studies by Mitchison [6] and Middlebrook and Cohn [7] established in animal models that some drug-resistant strains of tuberculosis were less pathogenic. Population-level molecular epidemiological studies support this finding. These studies estimate tuberculosis fitness by measuring the proportion of strains that are genetically clustered and attributable to recent transmission [8–10]. More recently, laboratory competitive fitness assays have demonstrated a variable fitness cost in drug-resistant *M*. *tuberculosis* bacilli, with most strains demonstrating a fitness cost and some demonstrating superior fitness [11–13]. However, studies of this kind do not account for the myriad of potential clinical, environmental, and socio-economic confounding variables that influence the ability of a patient to transmit the pathogen and cause tuberculosis disease in a contact. In vitro techniques also fail to measure fitness over the transmission cycle of the pathogen, from disease in the index case to disease in the contact.

Very few studies have estimated fitness in vivo by comparing the incidence of second cases of tuberculosis among contacts of patients with multidrug-resistant tuberculosis (MDRTB) to that among contacts of patients with drug-susceptible tuberculosis. Studies that have measured the incidence of second cases in households with MDRTB have lacked statistical power [14,15] or have not included drug-susceptible controls for comparison [16].

The aim of this prospective cohort study was to estimate the fitness of drug-resistant tuberculosis cases relative to drug-susceptible tuberculosis cases by determining the incidence of second cases of tuberculosis disease in households with an MDRTB index case relative to that in households with a drug-susceptible tuberculosis index case, while considering the effect of potential confounding variables.

## Methods

Ethical approval was obtained from the Institutional Review Board of Universidad Peruana Cayetano Heredia (IRB00001014) before the study began (approval number 57492). Institutional approval was also obtained from the Peruvian Ministry of Health and the regional tuberculosis control programs. Informed written consent was obtained from all study participants.

This 3-y prospective cohort study was undertaken between September 2010 and September 2013 in two study sites in metropolitan Lima (South Lima and Callao). The a priori alternative hypothesis was that the incidence of second cases of tuberculosis disease among the contacts of MDRTB index cases was different from that among the contacts of drug-susceptible tuberculosis index cases, independent of potentially confounding variables. Incidence rates of tuberculosis disease from previous studies [17,18] were used to perform a power calculation to determine the sample size for a detectable alternative hazard ratio (HR) with a power of 0.8. A minimum sample size of 800 MDRTB contacts and 1,600 drug-susceptible contacts (a 1:2 ratio) was determined to be needed to yield a significant difference between the two groups (two-tailed *p <* 0.05) with a HR ≤ 0.6 (S1 and S2 Texts; S1 Checklist).

### Field Methods

MDRTB index patients (resistant to at least rifampicin and isoniazid) and drug-susceptible index patients (susceptible to both rifampicin and isoniazid) were identified at diagnosis from each of the regional reference laboratories. Index patients were recruited at diagnosis (MDRTB or drug-susceptible tuberculosis), and a sputum culture was obtained. All tuberculosis patients in Peru are tested for human immunodeficiency virus (HIV); these data were available from the patient record. Patients who were able to expectorate sputum had their sputum tested by serial smear microscopy on a monthly basis. For the purposes of analyzing time to sputum conversion to negative during index case follow-up, index patients were regarded as becoming smear negative on the date of the first negative sample if they remained smear negative thereafter. Index patients were also regarded as being smear negative if they could no longer produce sputum and continued as such.

In order to minimize bias, for each MDRTB index patient recruited, at least two age- and sex-matched drug-susceptible controls were selected at random from the same study site as the index case for comparison. Following this, patients were visited at the health center and invited to participate in the study before providing written informed consent and completing a structured questionnaire at enrollment. The structured questionnaire recorded information on patient demographics, the household environment (crowding and house construction), and clinical data such as sputum smear result, culture results, and diagnosis dates. All variables used in the study questionnaire were field tested during a preliminary retrospective study [18].

Index patients were followed up at the local health center by study staff every 6 mo and asked about the well-being of family members and the occurrence of second cases of tuberculosis disease in the household. Any symptomatic contacts were encouraged to attend the local health post for testing. When recruitment to the study ended in January 2013, all index patients and their families were visited at home to ensure that all incident cases of tuberculosis disease during the study period had been recorded. The final round of active household-based follow-up for all families was designed to minimize potential bias from variable follow-up between MDRTB and drug-susceptible tuberculosis households. Chemoprophylaxis was prescribed and managed by the treating health center/physician in accordance with the national tuberculosis policy: national policy recommends chemoprophylaxis for the household contacts, <16 y of age, of patients with drug-susceptible tuberculosis and not for the household contacts of patients with MDRTB [19]. When there were delays in confirming MDRTB in the index case, MDRTB contacts below 16 y of age were given isoniazid chemoprophylaxis; however, after the MDRTB diagnosis was confirmed, the chemoprophylaxis was stopped. The number of contacts taking chemoprophylaxis and the duration of chemoprophylaxis were recorded for both groups.

### Laboratory Methods

Drug susceptibility testing for rifampicin and isoniazid was performed for all samples at the two regional reference laboratories (one located in Callao and one in South Lima) using the microscopic observation drug susceptibility assay (MODS) [20,21]. In accordance with the policy of the national tuberculosis program, samples for which MODS culture and direct drug susceptibility testing indicated drug resistance to rifampicin and isoniazid were subsequently tested at the national reference laboratory to confirm resistance to these drugs and to perform extended first- and second-line drug susceptibility testing using the proportions method. Extended first- and second-line drug susceptibility tests included ethambutol, streptomycin, ethionamide, kanamycin, capreomycin, ciprofloxacin, para-aminosalicylic acid (PAS), and cycloserine. The Wayne method was used for pyrazinamide drug susceptibility testing. Samples that were susceptible to both rifampicin and isoniazid were not sent for further second-line drug susceptibility testing.

Positive sputum cultures from index cases and contacts (when available) were sub-cultured on solid Ogawa medium and transported to the laboratories of Universidad Peruana Cayetano Heredia for DNA extraction and spoligotyping by conventional methodology [22].

### Data Analysis

Household contacts were defined as any person living in the same house as the index case for more than one day a week. Follow-up time for MDRTB and drug-susceptible tuberculosis household contacts started at the time of diagnosis of index case MDRTB and drug-susceptible tuberculosis, respectively. An “event” was defined as the development of tuberculosis disease in a household contact that occurred after the diagnosis of tuberculosis in the index case. Tuberculosis disease in household contacts was defined as any patient that had evidence of tuberculosis disease from sputum smear, culture, chest X-ray, or clinical diagnosis that led to initiation of anti-tuberculous treatment. This definition was chosen in order to include children who were diagnosed with tuberculosis disease and started on anti-tuberculous treatment by the treating physician without a microbiologically confirmed diagnosis. Contacts were censured if they were lost to follow-up. Contacts were regarded as lost to follow-up if they could not be located, had left the home, or had died (not as a consequence of tuberculosis). The incidence of tuberculosis disease was calculated as the number of incident cases of tuberculosis divided by the total number of contact follow-up person-years. Odds ratios (ORs) of the differences between the two comparison groups were calculated using the STATA csi command for ORs in cohort studies with an exact *p*-value. Previous tuberculosis disease was defined as any disease episode in which anti-tuberculous treatment was successfully completed more than 6 mo prior to the onset of the present tuberculosis episode. Missing data were minimized by revisiting households and health centers and cross-checking interview data against medical records. Missing values were then treated as “missing at random” using Stata’s default “listwise” deletion when included in multivariate regression analysis. The study database was deposited in the Dryad Digital Repository [23].

Details on diagnosis and treatment dates were also recorded for “co-prevalent” household contacts who had initiated treatment for tuberculosis disease before the diagnosis of the index case but who had not completed tuberculosis treatment by at least 6 mo prior to the diagnosis of the index case. Screening for co-prevalent cases was undertaken at the initial index case interview using the structured questionnaire, and this was checked again at subsequent household visits. Any co-prevalent case identified at initial screening was followed up as a household contact of the index case and was screened for signs and symptoms of tuberculosis at follow-up visits.

### Investigation of Factors Associated with Incident Disease in Contacts

Independent predictors of second cases were determined using a multivariate Cox regression survival analysis clustered at the level of the household. Each variable was tested for potential violation of the proportional hazards assumption by minus log-log plots and examination of the Schoenfeld residuals. Correction for clustering was undertaken at the level of the household to account for the correlation of variance within households. The STATA cluster clustvar command was used as the clustering method to provide a robust estimate of the standard error according to the Huber/White/Sandwich estimate of variance [24]. Known confounding variables identified a priori (specifically HIV status of contacts, contact age, contact sex, sputum smear status of the index case, and socio-economic status of the household), together with variables found to be *p <* 0.2 on univariate analysis, were included in the multivariate regression. The interactions between age and chemotherapy use, diabetes and index case drug resistance status, socio-economic status and index case sputum smear grade, contact employment and socio-economic status, and index case genotype and index case drug resistance status were all examined for significance in predicting second household cases of disease. A *p*-value of <0.05 was considered statistically significant in the multivariate regression. Analysis was undertaken using Stata (release 11, StataCorp). The preplanned analysis did not differ from the final analysis other than consideration of the potentially confounding association between index case genotype and second cases of tuberculosis disease, which was undertaken after reviewers’ comments.

## Results

### Study Recruitment

A total of 306 MDRTB index patients were identified for interview from the regional reference laboratories. Ninety-three MDRTB index patients could not be recruited: 45 patients (48% of unrecruited patients) could not be located either at the health post or at home as an erroneous address had been provided or they had abandoned treatment at the health post after having left a diagnostic specimen, 20 (22%) died before an interview could be undertaken, 16 (17%) were imprisoned, and 12 (13%) chose not to consent to enter the study. This left the household contacts of 213 newly diagnosed MDRTB index cases who were followed up as part of the study.

**Fig 1:**
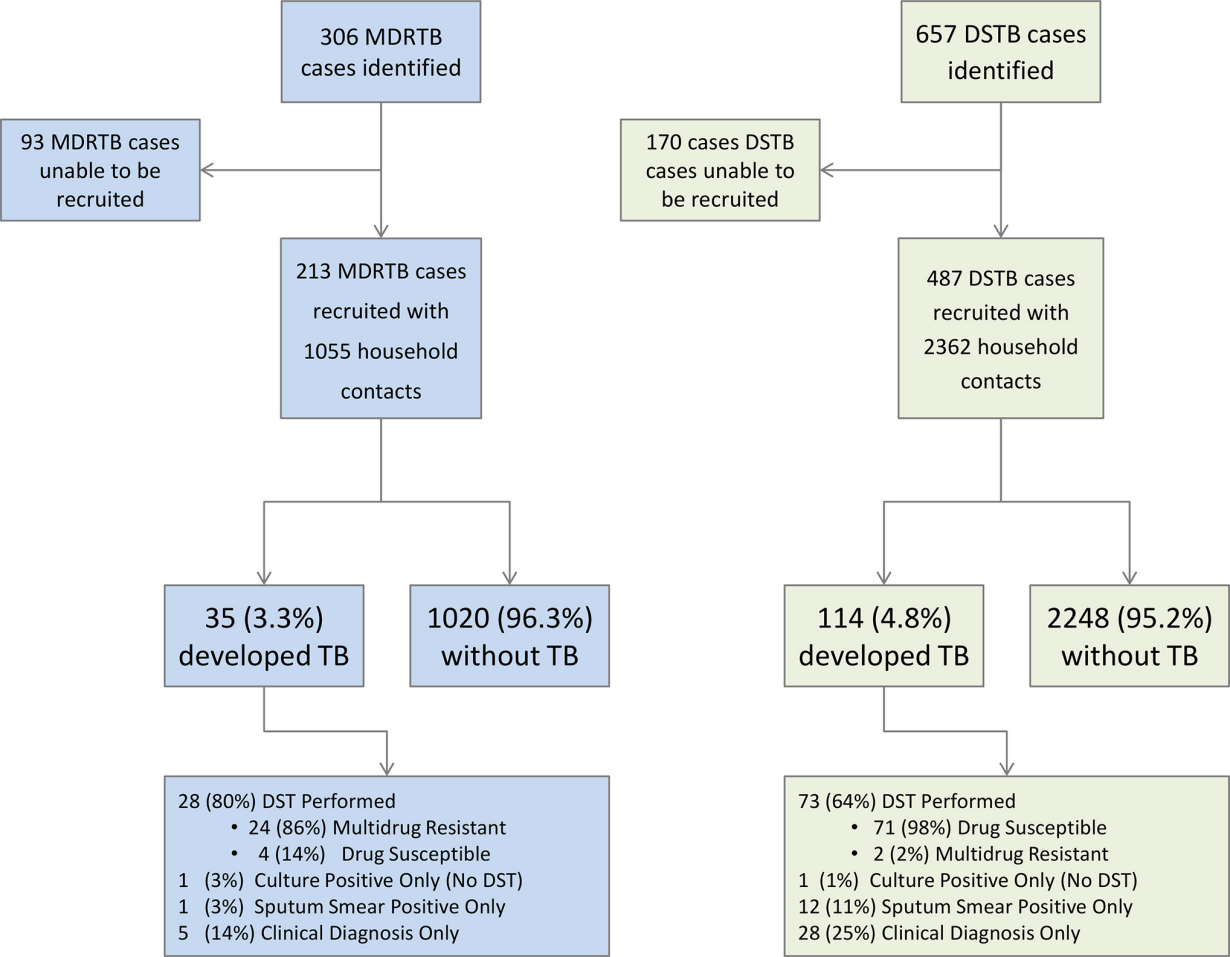
Flow diagram of recruitment to the study and study outcomes. DST, drug susceptibility testing; DSTB, drug-susceptible tuberculosis; TB, tuberculosis.

A total of 657 drug-susceptible tuberculosis index patients were identified as matched controls for the MDRTB index patients, of whom 170 could not be recruited: 147 patients (86%) could not be located either at the health post or at home as an erroneous address had been provided or they had abandoned treatment at the health post after leaving a diagnostic specimen, 20 patients (12%) chose not to consent to enter the study, and three patients (2%) died prior to interview. This left the household contacts of 487 newly diagnosed, matched drug-susceptible control index cases who were followed up as part of the study. MDRTB index cases lived with 1,055 household contacts (mean of 5.0 contacts per MDRTB index case), and drug-susceptible tuberculosis index cases lived with 2,362 household contacts (mean of 4.9 contacts per drug-susceptible tuberculosis index case) (Fig 1).

### Characteristics of Eligible Unrecruited Patients

The mean age of those recruited was not significantly different from the mean age of those not recruited (34 y for recruited patients and 33 for unrecruited patients, *p* = 0.28, non-paired Student’s *t* test), nor was the likelihood of smear positivity different (90% of recruited patients and 90% of unrecruited patients). However unrecruited patients were more likely to be male (72% of unrecruited patients and 61% of recruited patients, OR = 1.66, 95% CI 1.23–2.66, *p* = 0.001) and more likely to have previously received treatment (29% of unrecruited patients and 20% of recruited patients, OR = 1.61, 95% CI 1.16–2.25, *p* = 0.006).

### Incident Tuberculosis Disease in Contacts

Thirty-five second cases of tuberculosis disease occurred among the household contacts of MDRTB patients (35/1,055, 3.3%, 95% CI 2.3%–4.6%). This simple proportion (i.e., not accounting for follow-up time in both groups) was less than the proportion of household contacts of drug-susceptible tuberculosis index cases who developed tuberculosis disease (114/2,362, 4.8%, 95% CI 4.0%–5.8%), but the difference was of borderline statistical significance (OR = 0.68, 95% CI 0.46–0.99, *p* = 0.046).

Among the incident cases in MDRTB households that had a drug susceptibility test performed, 86% (95% CI 67%–96%, 24/28) also had MDRTB. Among the incident cases in drug-susceptible households that had a drug susceptibility test performed, 98% (95% CI 90.1%–99.7%, 71/73) also had drug-susceptible tuberculosis.

**Fig 2:**
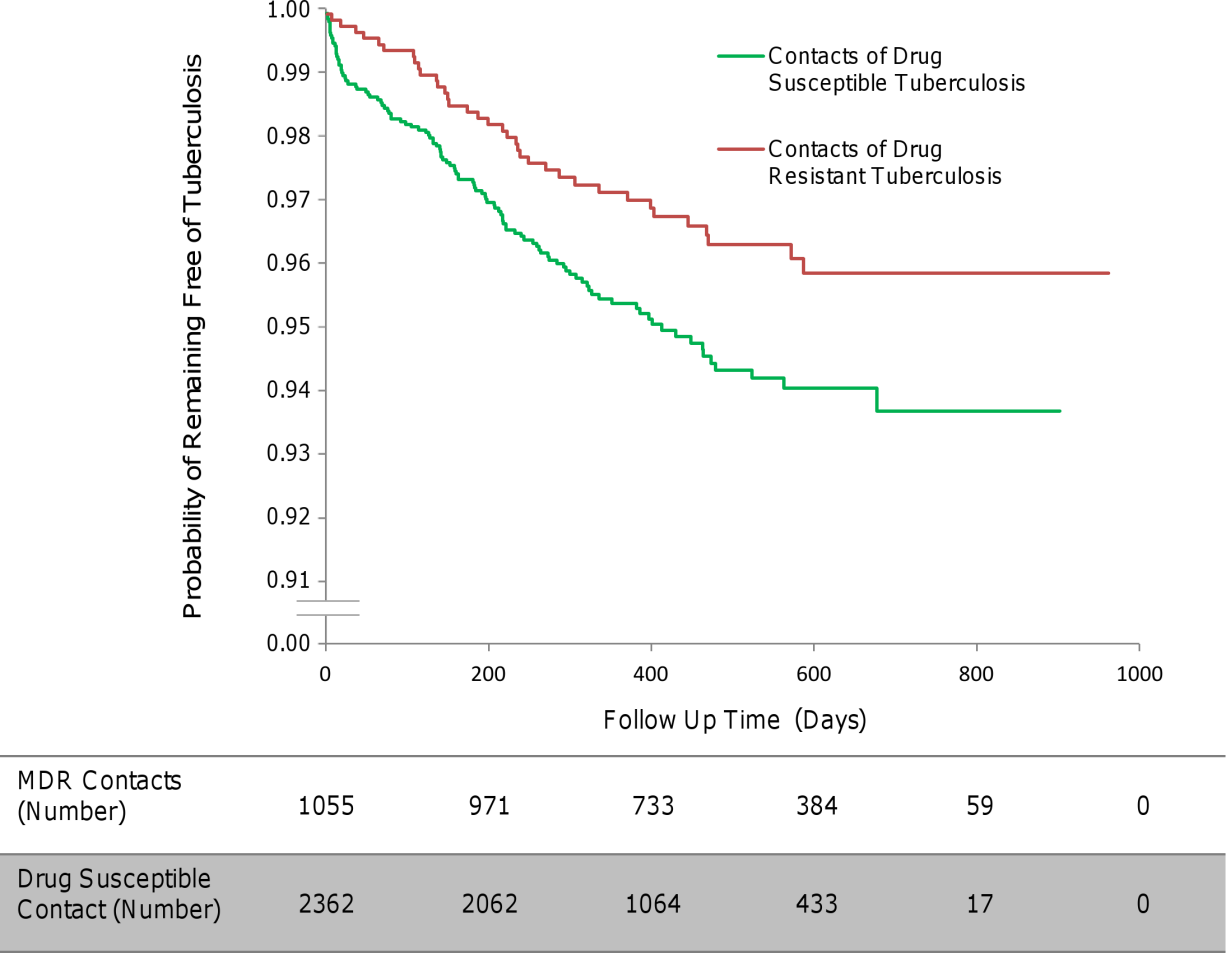
The incidence of second cases of tuberculosis disease in household contacts stratified by index case drug resistance.

The total follow-up time of MDRTB contacts was 1,425 person-years (mean follow-up time per MDRTB contact 494 d, standard deviation 199 d), during which 35 second cases arose, equating to an incidence of 2,456 per 100,000 contact follow-up person-years. The total follow-up time of drug-susceptible tuberculosis contacts was 2,620 person-years (mean follow-up time per drug-susceptible tuberculosis contact 406 d, standard deviation 189 d), during which 114 second cases arose, equating to an incidence of 4,351 per 100,000 contact follow-up person-years (multivariate analysis, HR 0.56, 95% CI 0.34–0.90, *p* = 0.017; Fig 2).

### Characteristics of Multidrug-Resistant and Drug-Susceptible Tuberculosis Index Cases and Contacts

Eight percent (18/213) of MDRTB index cases were HIV positive, while only 4% (20/487) of drug-susceptible tuberculosis index cases were HIV positive (OR = 2.25, 95% CI 1.18–4.30, *p* = 0.017; Table 1). MDRTB index patients were more likely to have had previous tuberculosis disease (68/213 [32%] for MDRTB and 62/487 [13%] for drug-susceptible tuberculosis, OR = 3.21, 95% CI 2.17–4.75, *p <* 0.001), to be hospitalized with their disease (39/213 [18%] for MDRTB and 50/487 [10%] for drug-susceptible tuberculosis, OR = 1.96, 95% CI 1.25–3.08, *p* = 0.004), and to describe any side effects of medication (145/213 [68%] for MDRTB and 206/487 [42%] for drug-susceptible tuberculosis, OR = 2.90, 95% CI 2.07–4.08, *p <* 0.001). The proportion of unemployment was higher among MDRTB patients (131/213 [61%] for MDRTB and 247/487 [51%] for drug-susceptible tuberculosis, OR = 1.55, 95% CI 1.11–2.15, *p* = 0.009), although more MDRTB patients had completed secondary education (143/213 [66%] for MDRTB and 275/487 [56%] for drug-susceptible tuberculosis, OR = 1.59, 95% CI 1.13–2.22, *p* = 0.007). Sputum smear grade was not significantly different between MDRTB and drug-susceptible tuberculosis index patients.

**Table 1:** Demographic data for index cases by drug resistance status.

Characteristic	MDRTB Index Patients	Drug-Susceptible Tuberculosis Index Patients	OR (95% CI), *p*-Value
**Number**	213	487	—
**Median (mean) age (years)**	28 (32)	29 (33)	Matched^1^
**Percent male**	39%	39%	Matched^1^
**Socio-economic status (tertile)** ^**2**^			
1	77 (36%)	211 (43%)	OR = 0.74 (0.53–1.03), *p* = 0.08
2	73 (34%)	137 (28%)	OR = 1.33 (0.94–1.88), *p* = 0.11
3	63 (30%)	139 (29%)	OR = 1.05 (0.74–1.50), *p* = 0.67
**Completed secondary education**	143 (67%)	274 (56%)	OR = 1.59 (1.13–2.22), *p* = 0.007
**HIV positive**	18 (8%)	20 (4%)	OR = 2.25 (1.18–4.30), *p* = 0.02
**Sputum smear grade**			
0	26 (12%)	41 (8%)	OR = 1.51 (0.90–2.53), *p* = 0.13
1	52 (24%)	145 (30%)	OR = 0.76 (0.53–1.10), *p* = 0.17
2	47 (22%)	133 (27%)	OR = 0.75 (0.51–1.10), *p* = 0.16
3	82 (38%)	152 (31%)	OR = 1.38 (0.99–1.92), *p* = 0.07
Unknown/not done	6 (3%)	16 (3%)	OR = 0.84 (0.34–2.15), *p* = 0.82
**Employment status**			
Unemployed	131 (62%)	247 (51%)	OR = 1.55 (1.11–2.15), *p* = 0.01
Working	60 (28%)	175 (36%)	OR = 0.70 (0.49–0.99), *p* = 0.05
Student	22 (10%)	62 (13%)	OR = 0.79 (0.47–1.32), *p* = 0.34
Unknown	0	3 (<1%)	OR = 0.00 (0.00–2.93), *p* = 0.55
**History of incarceration**	14 (7%)	22 (5%)	OR = 1.48 (0.75–2.93), *p* = 0.27
**History of hospitalization** ^**3**^	39 (18%)	50 (10%)	OR = 1.96 (1.25–3.08), *p* = 0.004
**Median (mean) crowding (people per room)**	1.8 (2.1)	2 (2.2)	*p* = 0.24^4^
**History of previous tuberculosis disease**	68 (32%)	62 (13%)	OR = 3.21 (2.17–4.75), *p <* 0.001
**Any side effects of treatment**	145 (68%)	206 (42%)	OR = 2.90 (2.07–4.08), *p <* 0.001
**Median (mean) reported cough duration (weeks)**	4 (6.9)	4 (6.0)	*p* = 0.10^4^
**Alcohol use (more than one unit/day)**	26 (12%)	53 (11%)	OR = 1.14 (0.69–1.17), *p* = 0.61
**Tobacco use (any cigarettes each week)**	38 (18%)	70 (14%)	OR = 1.29 (0.84–1.99), *p* = 0.26
**Spoligotype family (SpolDB4 Database)**			
Haarlem	23 (11%)	120 (25%)	OR = 0.37 (0.23–0.60), *p <* 0.001
Beijing	19 (9%)	53 (11%)	OR = 0.80 (0.47–1.38), *p* = 0.50
LAM^5^	42 (20%)	50 (10%)	OR = 2.15 (1.38–3.35), *p <* 0.001
T	76 (36%)	67 (14%)	OR = 3.48 (2.38–5.08), *p <* 0.001
Other Euro-American^5^	10 (5%)	51 (10%)	OR = 2.23 (1.12–4.42), *p* = 0.02
Orphan/no family	17 (8%)	58 (11%)	OR = 1.49 (0.85–2.61), *p* = 0.18
Unknown (no data)	26 (12%)	88 (18%)	OR = 0.63 (0.40–1.01), *p* = 0.06

Data are number (percent) unless otherwise indicated.

^1^Drug-susceptible tuberculosis and MDRTB index cases were matched by sex and age.

^2^Socio-economic status was derived from the Necesidades Basicas Insatisfechas score, a locally validated scoring system used as part of the Peruvian National Census. This score allows a distinction to be made between different levels of poverty within a shanty town.

^3^Hospitalization due to present tuberculosis disease.

^4^Mann–Whitney U test, otherwise two sample test of proportions with exact *p*-values.

^5^LAM indicates Latin American Mediterranean. “Other Euro-American” includes strains from the S family and the X family and strains that were present in the SpolDB4 Database but had not been assigned a family yet.

The proportion of index patients with disease caused by the Haarlem spogliotype family was greater among drug-susceptible tuberculosis index patients (23/213 [11%] for MDRTB versus 120/487 [25%] for drug-susceptible tuberculosis, OR = 0.37, 95% CI 0.23–0.60, *p <* 0.001), while the Latin American Mediterranean spogliotype family (42/213 [20%] for MDRTB versus 50/487 [10%] for drug-susceptible tuberculosis, OR = 2.15 95% CI 1.38–3.35, *p <* 0.001) and T spogliotype family (76/213 [36%] for MDRTB versus 67/487 [14%] for drug-susceptible tuberculosis, OR = 3.48 95%, CI 2.38–5.08, *p <* 0.001) were overrepresented among MDRTB index cases.

At baseline, the contacts of MDRTB index patients were more likely to report a previous history of tuberculosis disease than the contacts of patients with drug-susceptible tuberculosis (302/1,055 [29%] for MDRTB and 281/2,362 [12%] for drug-susceptible tuberculosis, OR = 2.97, 95% CI 2.47–3.56, *p <* 0.001; Table 2). Five contacts of MDRTB index patients and ten contacts of drug-susceptible tuberculosis index patients were identified at the initial interview as being co-prevalent cases (5/1,055 for MDRTB versus 10/2,362 for drug-susceptible tuberculosis, OR = 0.79, 95% CI 0.40–3.14, *p* = 0.79). Among these patients, only one drug-susceptible tuberculosis contact developed tuberculosis disease (either relapse or new infection) during the course of the study. Fewer contacts of MDRTB patients had received isoniazid chemoprophylaxis (132/1,055 [12.5%] among MDRTB contacts and 407/2,362 [17.2%] among drug-susceptible tuberculosis contacts, OR = 0.69 95% CI 0.56–0.85, *p <* 0.001).

**Table 2:** Demographic data for household contacts by drug resistance status of the index case.

Characteristic	Contacts of Newly Diagnosed MDRTB Patients	Contacts of Newly Diagnosed Drug-Susceptible Tuberculosis Patients	OR (95% CI), *p*-Value
**Number of household contacts**	1,055	2,362	—
**Median (mean) Contacts per index case**	4 (5.0)	4 (4.9)	*p* = 0.69^1^
**Number of second cases of tuberculosis**	35 (3.3%)	114 (4.8%)	OR = 0.68 (0.46–0.99), *p* = 0.046
**Co-prevalent cases**	5 (0.5%)	10 (0.4%)	OR = 0.79 (0.40–3.14), *p* = 0.79
**Median (mean) contact age (years)**	25 (28)	25 (28)	*p* = 0.57^1^
**Age stratum**			
0–10 y	154 (15%)	346 (15%)	OR = 0.99 (0.81–1.22), *p* = 1.00
10–20 y	195 (18%)	430 (18%)	OR = 1.02 (0.84–1.23), *p* = 0.85
20–30 y	177 (17%)	425 (18%)	OR = 0.92 (0.76–1.11), *p* = 0.39
30–40 y	220 (21%)	453 (19%)	OR = 1.11 (0.93–1.33), *p* = 0.26
40–50 y	114 (11%)	271 (11%)	OR = 0.93 (0.74–1.18), *p* = 0.60
50–60 y	109 (10%)	220 (9%)	OR = 1.12 (0.88–1.43), *p* = 0.35
60–70 y	56 (5%)	118 (5%)	OR = 1.07 (0.77–1.48), *p* = 0.70
80–90 y	14 (1%)	57 (2%)	OR = 0.54 (0.30–0.97), *p* = 0.038
90–100 y	14 (1%)	33 (1%)	OR = 0.95 (0.51–1.76), *p* = 1.00
Unknown	2 (0.2%)	9 (0.2%)	OR = 0.50 (0.00–2.04), *p* = 0.52
**Male sex**	517 (49%)	1,181 (50%)	OR = 0.96 (0.83–1.11), *p* = 0.60
**Diabetic**	12 (1%)	29 (1%)	OR = 1.41 (0.69–2.88), *p* = 0.33
**Completed secondary education**	494 (47%)	1,046 (44%)	OR = 1.11 (0.96–1.28), *P* = 0.17
**HIV positive**	8 (0.8%)	12 (0.5%)	OR = 1.50 (0.63–3.57), *p* = 0.47
**Employment status**			
Unemployed	256 (24%)	569 (24%)	OR = 1.01 (0.85–1.20), *p* = 0.93
Working	420 (40%)	928 (39%)	OR = 1.02 (0.88–1.19), *p* = 0.79
Students	258 (24%)	625 (26%)	OR = 0.90 (0.76–1.06), *p* = 0.22
Unknown	121 (12%)	240 (10%)	OR = 1.15 (0.91–1.44), *p* = 0.25
**History of previous tuberculosis disease**	302 (29%)	281 (12%)	OR = 2.97 (2.47–3.56), *p <* 0.001
**Isoniazid chemoprophylaxis**			
Treated	132 (13%)	407 (17%)	OR = 0.69 (0.56–0.85), *p <* 0.001
Unknown	1 (0.1%)	11 (0.5%)	—
**Crowding (people per room)**			
1–2 per room	307 (29%)	680 (29%)	OR = 1.02 (0.87–1.19), *p* = 0.87
2–3 per room	365 (35%)	739 (40%)	OR = 1.17 (1.00–1.36), *p* = 0.048
>3 per room	376 (36%)	942 (40%)	OR = 0.83 (0.72–0.97), *p* = 0.020
Unknown	7 (0.6%)	1 (0.04%)	—
**Contact sleeping in the same room as the index case**	188 (19%)	452 (19%)	OR = 0.92 (0.76–1.11), *p* = 0.37

Data are number (percent) unless otherwise indicated.

^1^Mann–Whitney U test, otherwise two sample test of proportions with exact *p*-values.

### Independent Predictors of Second Cases amongst Household Contacts

#### Index case factors (multivariate analysis)

**Fig 3:**
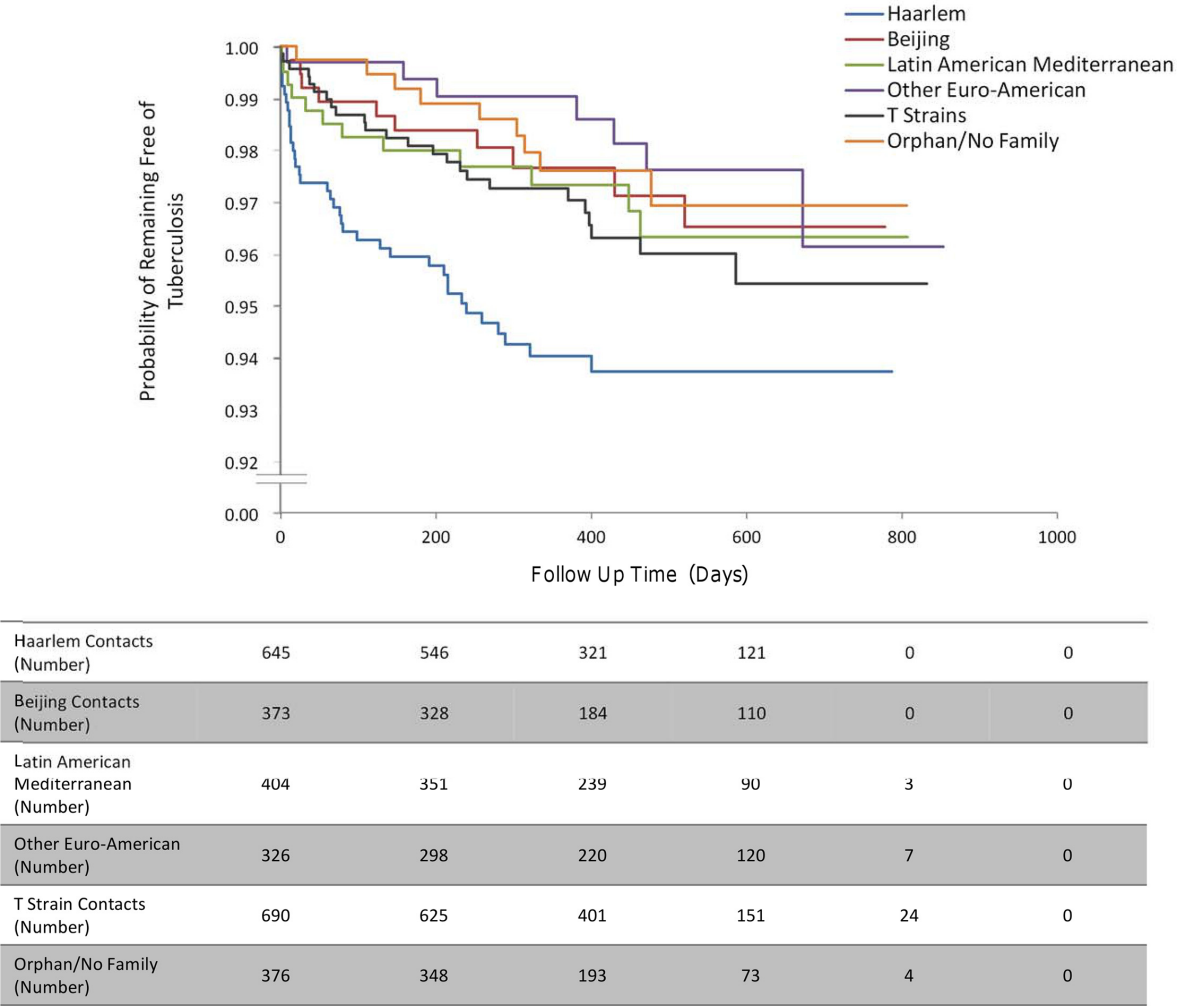
The incidence of second cases of tuberculosis disease stratified by index case genotype. “Other Euro-American” includes strains from the S family and the X family and strains that were present in the SpolDB4 Database that had not been assigned a family yet.

**Fig 4:**
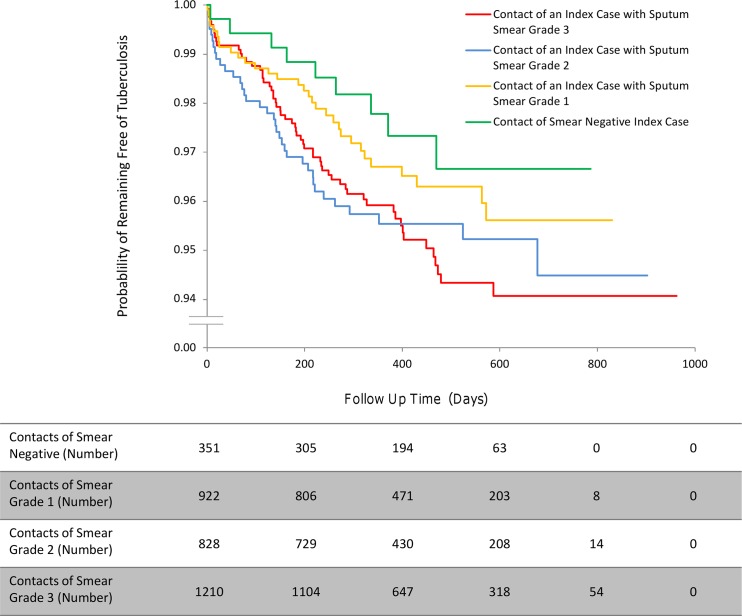
The incidence of second cases of tuberculosis disease stratified by index case sputum smear grade.

The incidence of second cases of tuberculosis disease among the household contacts of MDRTB patients was lower than that among the contacts of drug-susceptible tuberculosis index patients (HR 0.56, 95% CI 0.34–0.90, *p* = 0.017; Fig 2). This finding remained statistically significant independent of potentially confounding variables in the context of a clustered multivariate Cox regression survival analysis. Relative to the Haarlem spogliotype family, index cases who had disease caused by a Latin American Mediterranean strain (HR 0.78, 95% CI 0.37–1.62, *p* = 0.51) or a T strain (HR 0.67, 95% CI 0.34–1.32, *p* = 0.25) were not associated with a greater incidence of second cases. However, other Euro-American strains (HR 0.18, 95% CI 0.06–0.51, *p* = 0.001) and the Beijing spogliotype family (HR 0.43, 95% CI 0.20–0.92, *p* = 0.031) gave rise to fewer second cases of tuberculosis disease among contacts (Fig 3). As the index sputum smear grade increased, the HR of disease in contacts also increased, although this association did not reach statistical significance in the multivariate regression (Table 3; Fig 4). No interaction terms between index, contact, and household variables were identified as statistically significant.

**Table 3:** Index case predictors of tuberculosis disease in all household contacts (both multidrug-resistant tuberculosis contacts and drug-susceptible tuberculosis contacts).

Index Case Characteristic	Univariate HR (95% CI), *p*-Value	Multivariate HR (95% CI), *p*-Value
**Age**		
10–20 y	1.09 (0.56–2.14), *p* = 0.78	—
20–30 y	0.99 (0.52–1.89), *p* = 0.98	—
30–40 y	0.80 (0.39–1.68), *p* = 0.56	—
40–50 y	0.99 (0.46–2.12), *p* = 0.97	—
50–60 y	Reference level	—
60–70 y	0.16 (0.02–1.21), *p* = 0.08	—
70–80 y	1.13 (0.25–5.11), *p* = 0.87	—
80–90 y	2.27 (0.50–10.26), *p* = 0.29	—
**Male sex**	1.14 (0.82–1.59), *p* = 0.44	—
**Employment status**		
Unemployed	Reference level	—
Working	0.97 (0.67–1.39), *p* = 0.86	—
Student	1.29 (0.82–2.02), *p* = 0.27	—
**Secondary education completed** ^**1**^	0.61 (0.44–0.86), *p* = 0.004	0.70 (0.45–1.08), *p* = 0.24
**Sputum smear grade at tuberculosis diagnosis** ^**1**^		
0	Reference level	—
1	1.26 (0.60–2.66), *p* = 0.54	0.96 (0.47–1.96), *p* = 0.92
2	1.72 (0.83–3.57), *p* = 0.15	1.03 (0.51–2.05), *p* = 0.94
3	1.83 (0.91–3.67), *p* = 0.09	1.33 (0.73–2.43), *p* = 0.35
**History of previous tuberculosis disease**	0.79 (0.49–1.25), *p* = 0.31	—
**HIV positive**	1.05 (0.52–2.14), *p* = 0.89	—
**Duration of cough (weeks)**	0.99 (0.97–1.02), *p* = 0.65	—
**Diabetes** ^**1**^	0.21 (0.05–0.85), *p* = 0.029	0.20 (0.06–0.74), *p* = 0.016
**MDRTB** ^**1**^	0.62 (0.42–0.90), *p* = 0.012	0.56 (0.34–0.90), *p* = 0.017
**History of incarceration**	1.25 (0.64–2.47), *p* = 0.50	—
**Hospitalization (due to present illness)**	0.97 (0.60–1.55), *p* = 0.89	—
**Alcohol use (>1 unit/day)**	1.29 (0.80–2.09), *p* = 0.30	—
**Tobacco use (any use/week)**	0.91 (0.57–1.46), *p* = 0.70	—
**Side effects of medication**	0.92 (0.66–1.29), *p* = 0.65	—
**Spoligotype family** ^**1**^ **(SpolDB4 Database)**		
Haarlem	Reference level	Reference level
Beijing	0.44 (0.22–0.89), *p* = 0.023	0.43 (0.20–0.92), *p* = 0.031
LAM^2^	0.50 (0.26–0.96), *p* = 0.037	0.78 (0.37–1.62), *p* = 0.51
T	0.57 (0.34–0.95), *p* = 0.034	0.67 (0.34–1.32), *p* = 0.25
Other Euro-American^2^	0.33 (0.15–0.74), *p* = 0.007	0.18 (0.06–0.51), *p* = 0.001
Orphan/no family	0.39 (0.19–0.81), *p* = 0.012	0.45 (0.19–1.08), *p* = 0.08
Unknown (no data)	1.47 (0.96–2.26), *p* = 0.07	1.61 (0.90–2.91), *p* = 0.11

^1^These variables were included in the multivariate regression as they were determined to be *p <* 0.2 on univariate analysis or were known confounding variables identified a priori that have previously been associated with second cases of tuberculosis.

^2^LAM indicates Latin American Mediterranean. “Other Euro-American” includes strains from the S family and the X family and strains that were present in the SpolDB4 Database but had not been assigned a family yet.

#### Contact factors (multivariate analysis)

**Fig 5:**
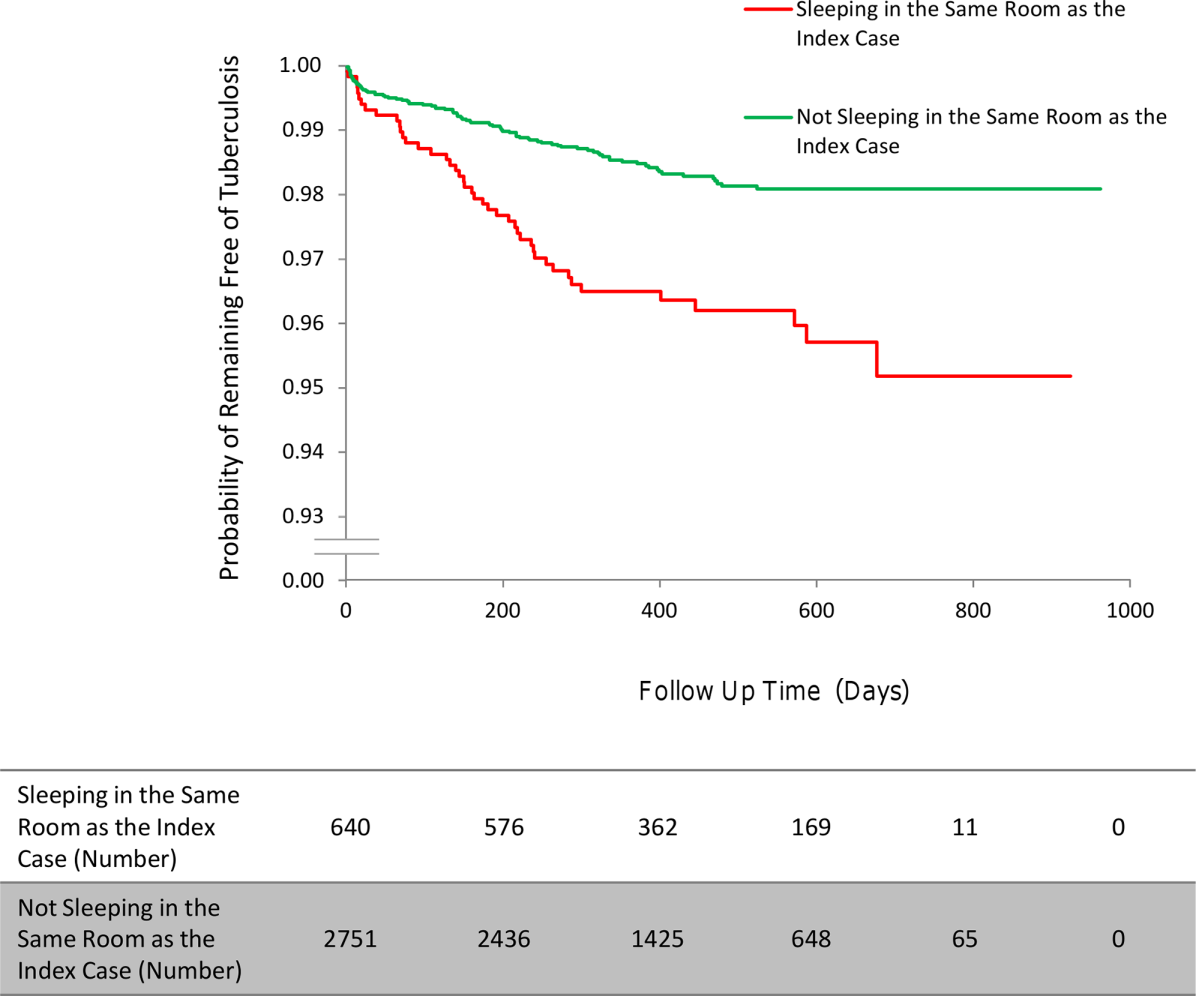
The incidence of second cases of tuberculosis disease stratified by a household contact sleeping/not sleeping in the same room as the index case.

Household contacts who shared a sleeping room with the index case had a higher incidence of tuberculosis disease than household contacts sleeping in a different room from the index case (HR 1.76, 95% CI 1.15–2.69, *p* = 0.009; Fig 5). Household contacts with diabetes had a higher incidence of disease than those without diabetes (HR 5.49, 95% CI 1.96–15.39, *p* = 0.001), as did male household contacts compared to female contacts (HR 1.92, 95% CI 1.21–3.02, *p* = 0.005), contacts with a previous history of tuberculosis disease compared to those with no previous tuberculosis disease (HR 2.47, 95% CI 1.41–4.34, *p* = 0.002), and contacts with HIV compared to contacts without HIV (HR 3.98, 95% CI 1.34–11.87, *p* = 0.013). Contacts who received isoniazid chemoprophylaxis after exposure were significantly less likely to develop tuberculosis disease than those who did not receive isoniazid chemoprophylaxis (HR 0.04, 95% CI 0.01–0.30, *p* = 0.002). Household contacts who were employed were less likely to develop tuberculosis disease than unemployed household contacts (HR 0.47, 95% CI 0.28–0.78, *p* = 0.004; Table 4).

**Table 4:** Contact predictors of tuberculosis disease in all household contacts (both multidrug-resistant tuberculosis contacts and drug-susceptible tuberculosis contacts).

Contact Characteristic	Univariate HR (95% CI), *p*-Value	Multivariate HR (95% CI), *p*-Value
**Age** ^**1**^		
0–10 y	0.55 (0.30–1.02), *p* = 0.06	0.82 (0.24–2.75), *p* = 0.74
10–20 y	1.05 (0.62–1.78), *p* = 0.84	1.57 (0.64–3.85), *p* = 0.32
20–30 y	1.55 (0.96–2.50), *p* = 0.07	1.91 (1.06–3.45), *p* = 0.031
30–40 y	Reference level	—
40–50 y	0.73 (0.38–1.40), *p* = 0.34	0.90 (0.41–1.96), *p* = 0.79
50–60 y	0.30 (0.11–0.77), *p* = 0.013	0.33 (0.11–1.03), *p* = 0.06
60–70 y	0.44 (0.15–1.27), *p* = 0.13	0.49 (0.15–1.58), *p* = 0.23
**Male sex** ^**1**^	1.48 (1.07–2.05), *p* = 0.018	1.92 (1.21–3.02), *p* = 0.005
**Employment status**		
Unemployed^**1**^	Reference level	—
Working	0.69 (0.47–1.03), *p* = 0.07	0.47 (0.28–0.78), *p* = 0.004
Student	0.72 (0.46–1.12), *p* = 0.15	0.78 (0.38–1.62), *p* = 0.51
**Secondary education completed**	1.11 (0.79–1.58), *p* = 0.53	—
**HIV positive** ^**1**^ ^,^ ^**2**^	8.99 (4.21–19.20), *p <* 0.001	3.98 (1.34–11.87), *p* = 0.013
**Diabetes** ^**1**^ ^,^ ^**2**^	3.52 (1.55–7.97), *p* = 0.002	5.49 (1.96–15.39), *p* = 0.001
**Sleeping in the same room as the index case** ^**1**^	2.29 (1.61–3.26), *p <* 0.001	1.76 (1.15–2.69), *p* = 0.009
**History of previous tuberculosis disease** ^**1**^ ^,^ ^**3**^	2.83 (1.83–4.39), *p <* 0.001	2.47 (1.41–4.34), *p* = 0.002
**History of taking chemoprophylaxis** ^**1**^ ^,^ ^**3**^	0.11 (0.03–0.34), *p <* 0.001	0.04 (0.01–0.30), *p* = 0.002

^1^These variables were included in the multivariate regression as they were determined to be *p <* 0.2 on univariate analysis or were known confounding variables identified a priori that have previously been associated with second cases of tuberculosis.

^2^Contacts who had not been tested for HIV/diabetes were assumed to be negative.

^3^These variables were identified as being time-varying covariates with increasing hazards over the length of the study; the corresponding HR should therefore be regarded as an average over the follow-up period.

#### Household factors (multivariate analysis)

**Fig 6:**
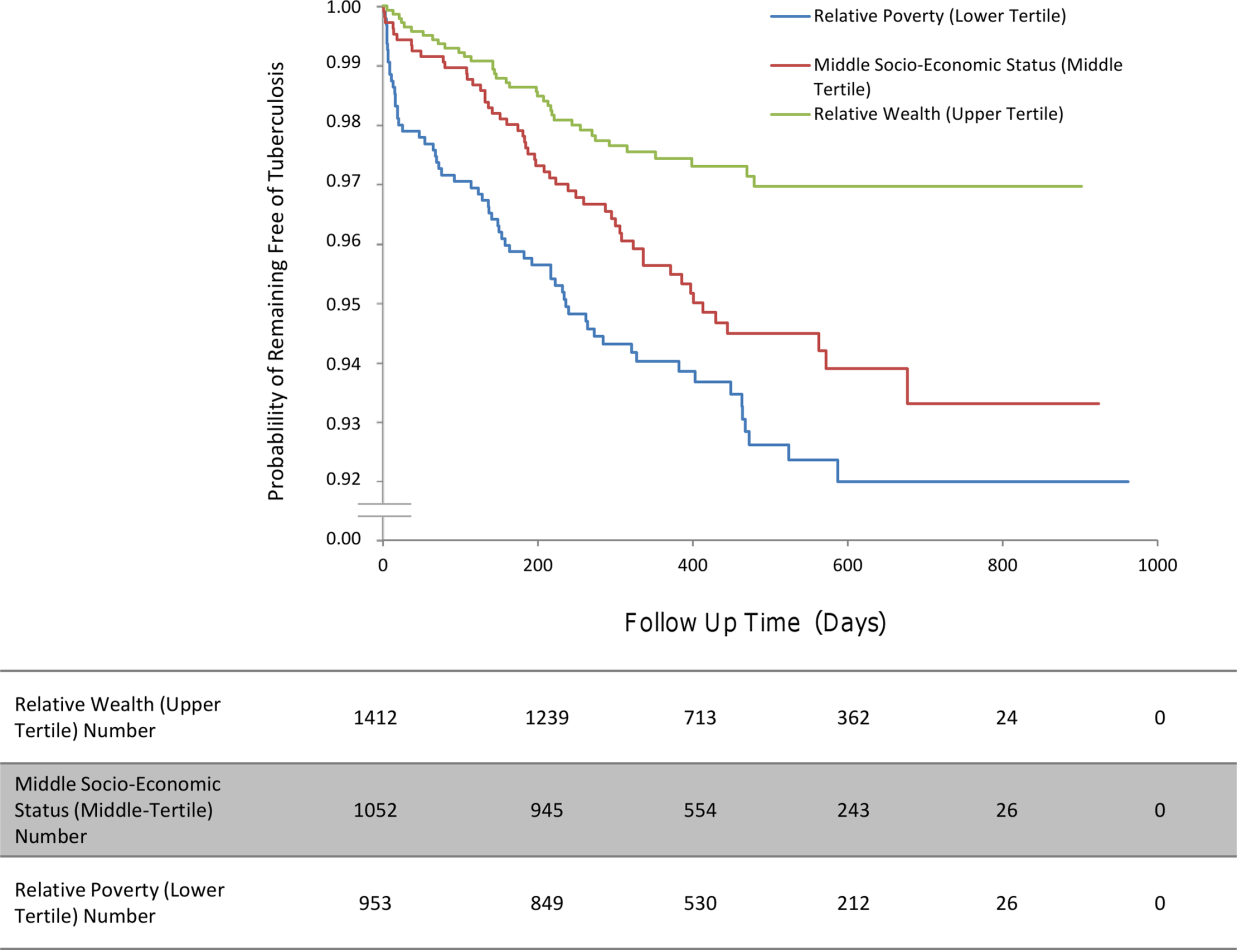
The incidence of second cases of tuberculosis disease stratified by household socio-economic status.

Second cases of tuberculosis disease occurred more often in households of the lowest socio-economic tertile relative to the highest socio-economic tertile (HR 2.86, 95% CI 1.60–4.76, *p <* 0.001; Fig 6). However, crowding was not significantly associated with an increased incidence of second cases (Table 5).

**Table 5:** Socio-demographic predictors of tuberculosis disease in contacts.

Household Characteristic	Univariate HR (95% CI), *p*-Value	Multivariate HR (95% CI), *p*-Value
**Socio-economic status (tertiles)** ^**1**^		
1	Reference level	—
2	1.87 (1.22–2.87), *p <* 0.01	1.65 (0.92–2.98), *p* = 0.10
3	2.76 (1.74–3.94), *p <* 0.01	2.86 (1.60–4.76), *p <* 0.001
**Crowding (people per room)**		
1–2 per room	Reference level	—
2–3 per room	0.88 (0.58–1.33), *p* = 0.55	—
>3 per room	0.97 (0.66–1.43), *p* = 0.88	—

^1^This variable was included in the multivariate regression as it was determined to be *p <* 0.2 on univariate analysis or was a known confounding variable identified a priori that has previously been associated with second cases of tuberculosis.

When diagnostic delay (between the date of the initial sputum sample and the start date of appropriate treatment) was included in the multivariate regression, the HR for the association of index case drug resistance status with the incidence of second cases among household contacts decreased from 0.56 to 0.52, and the *p*-value decreased from 0.017 to 0.008. However, this analysis was not chosen as the primary analysis as it risked including diagnostic delay time for some secondary MDRTB patients while they were initially treated as having drug-sensitive tuberculosis and therefore losing discrimination between the groups.

#### Outcomes of Multidrug-Resistant and Drug-Susceptible Tuberculosis Index Cases

Ninety-six percent (467/487) of drug-susceptible tuberculosis and 95% (202/213) of MDRTB index patients provided sputum samples for analysis. Of these, 80% (375/467) of drug-susceptible tuberculosis index patients had either converted to sputum smear negative or were unable to expectorate sputum within the first 8 wk of starting anti-tuberculous treatment. However, only 52% (106/202) of MDRTB index cases had converted to smear negative or were unable to expectorate sputum in the same time period (OR = 0.27, 95% CI 0.19–0.39, *p <* 0.001). Continued sputum smear positivity 6 mo into tuberculosis treatment was noted for 13% (27/202) and 4% (18/467) of MDRTB and drug-susceptible tuberculosis index cases, respectively (OR = 3.85, 95% CI 2.08–7.11, *p <* 0.001).

A greater proportion of index cases with MDRTB died as a consequence of their disease (9/213 [4.2%] for MDRTB versus 3/487 [0.6%] for drug-susceptible tuberculosis, OR = 7.12, 95% CI 2.06–24.53, *p <* 0.001), abandoned treatment (19/213 [8.9%] for MDRTB versus 24/487 [4.9%] for drug-susceptible tuberculosis, OR = 1.99, 95% CI 1.07–3.70, *p* = 0.037), or had their treatment regime changed as a consequence of a drug susceptibility test (69/213 [32.4%] for MDRTB versus 3/487 [0.6%] for drug-susceptible tuberculosis, OR = 77.3, 95% CI 25.36–235.14, *p <* 0.001).

## Discussion

This prospective cohort study has demonstrated that over 3 y of follow-up the incidence of tuberculosis disease in households with an MDRTB index case is almost half that of households with a drug-susceptible tuberculosis index case. This finding remained statistically significant even after considering confounding clinical and socio-demographic variables and despite the fact that index patients with MDRTB were sputum smear positive for longer.

The HR is a measure of the relative reproductive fitness of drug-resistant as compared to drug-susceptible tuberculosis. Most studies of the fitness of drug-resistant *M*. *tuberculosis* bacilli have been undertaken in animal models [6,7,25] or using competitive fitness assays in the laboratory [11–13]; however, these studies do not incorporate the potential clinical, environmental, and socio-economic confounding variables that influence the ability of an index patient to cause a second case of disease in the community. Despite this, our findings support the evidence from these studies undertaken in vitro and suggest that in households, at least during the first 3 y following exposure, MDRTB patients are less able to cause secondary disease in contacts than patients with drug-susceptible tuberculosis.

Our findings are also in keeping with estimates of drug-resistant tuberculosis fitness from molecular epidemiological studies. Drug-resistant strains in these studies were less associated with genetic clustering and therefore were considered to contribute less to recent transmission [8–10]. These studies benefit from a population-level design, as they are able to include transmission (as determined by molecular clustering) both in the community and in the household. However, clustering studies, particularly in low-incidence regions, are complicated by the relatively recent emergence or importation of drug-resistant strains. The fitness of recently emerging drug-resistant strains may be overestimated as isolated strains from latent reactivation will not yet have arisen. Equally, drug-resistant tuberculosis fitness may be underestimated if there is proportionally more drug resistance among recently imported unclustered strains.

The future global spread of MDRTB is very dependent on the relative fitness of drug-resistant and drug-susceptible bacilli. Mathematical models predict that the greater the relative fitness of drug-resistant tuberculosis, the greater the size of the drug-resistant epidemic [3,4]. This makes our finding welcome and encouraging news for tuberculosis control programs and health services attempting to contain the spread of MDRTB. The most recently published survey by the World Health Organization [26], in October 2014, supports our findings, demonstrating that globally the proportion of new cases of MDRTB did not change between 2008 and 2013, remaining at 3.5% of new cases. However, these findings do not preclude the future emergence and selection of fitter multidrug-resistant strains that would make the control of MDRTB increasingly difficult. Fitness is therefore time, bacterial strain, and place dependent. The fitness of MDRTB estimated here must also be taken in the context of the national tuberculosis control program, the household follow-up study design, and the study setting. This context may limit the extrapolation of these findings to other countries, particularly when considering outbreaks in the community or prisons, where conditions may favor the spread of MDRTB. Contacts outside the house may come into contact with and be infected by the index case at any stage during the infectious period (which, because of delays in diagnosis, is longer for MDRTB patients), while contacts inside the house, because of frequent exposure, are more likely to be infected earlier in the infectious period. This factor could increase the number of second cases for MDRTB, and hence MDRTB fitness estimates, in the community.

The incidence rate of disease among MDRTB contacts in this study was almost identical to our previous estimate of the incidence of disease in MDRTB contacts established in a preliminary retrospective study [18], and the disease yield among drug-susceptible tuberculosis contacts in this study was very similar to that reported elsewhere [17]. In a smaller study that identified six diseased contacts of MDRTB patients and 11 diseased contacts of drug-susceptible tuberulosis patients in Brazil, Teixeira et al. established that the proportion of second cases of tuberculosis disease was the same in both groups. [14]. This study did not have the statistical power to detect a difference between the number of second cases in MDRTB households and drug-susceptible tuberculosis households, nor did it make a formal assessment of the incidence in terms of a survival analysis.

We found that the incidence of second cases of tuberculosis disease was significantly higher in households with the lowest socio-economic status, among male contacts between 20–30 y old, and among contacts who shared a sleeping room with the index case. As expected, those household contacts with HIV or diabetes also had a significantly higher incidence of tuberculosis disease. The incidence of tuberculosis disease among the contacts of index patients with disease caused by the Beijing strain of tuberculosis was significantly lower than among those exposed to disease caused by the Haarlem strain. This is in contrast to studies in the former Soviet states [27] and suggests that the Beijing strain is no more virulent than other strains in the South American population we studied. The incidence of disease was decreased among contacts who were employed, which may be a consequence of decreased exposure to the index case. Chemoprophylaxis also significantly decreased the incidence of disease among contacts, despite the fact that many of the household contacts were those of MDRTB patients. The low incidence of disease among contacts aged 0–10 y may be a consequence of the low prevalence of HIV in this setting and/or the effectiveness of chemoprophylaxis in preventing drug-susceptible tuberculosis in this group.

Our study has a number of important strengths and limitations. Following a large cohort of MDRTB patients over 3 y—during the highest risk period for incident tuberculosis disease following acquisition of new infection—enabled us to recruit enough newly diagnosed patients to accurately compare the incidence of disease in both groups with sufficient statistical power to detect a difference between the two groups. Comprehensive index patient interviews gave us detailed data on potentially confounding clinical, demographic, and socio-economic variables, while active case finding visits to the households maximized the sensitivity of case detection. Relative to drug-susceptible tuberculosis index patients, more MDRTB index patients died before they could be recruited to the study. These patients could have harbored MDRTB strains of greater transmissibility and therefore increased the number of second cases among MDRTB households. Genotyping culture-positive contacts would have allowed us to be more certain of the relative contribution of extra-domiciliary transmission to MDRTB and drug-susceptible tuberculosis households [28]. However, drug-susceptible tuberculosis control patients were selected from the same region as MDRTB index cases, and their household contacts were therefore likely to be exposed to a similar risk of tuberculosis infection from the surrounding community.

The duration of this study was over 3 y; however, latent tuberculosis infection may reactivate decades after infection has occurred [29]. To study a cohort of patients for this length of time would require excessive resources, and the reactivation of latent infection would influence the findings of the study only if patients latently infected with drug-resistant tuberculosis reactivated at different rates than those latently infected with drug-susceptible tuberculosis.

In summary, this study has demonstrated that in the first 3 y following exposure to the index case, the incidence of secondary cases of tuberculosis disease is greater among the household contacts of drug-susceptible tuberculosis patients than among those of MDRTB patients. This suggests that MDRTB is less fit (less transmissible and/or less able to cause disease) than drug-susceptible tuberculosis, at least in households. The fitness of MDRTB relative to drug-susceptible tuberculosis is one of the most important determinants of future MDRTB spread. A low relative fitness of MDRTB improves the chances of containing and diminishing the spread of drug-resistant tuberculosis.

## Supporting Information

S1 Checklist The study STROBE checklist. (DOCX)

S1 Text The study institutional review board approval and protocol. (PDF)

S2 Text Extended study protocol. (PDF)

## Abbreviations:

HR: hazard ratio
MDRTB: multidrug-resistant tuberculosis
MODS: microscopic observation drug susceptibility assay
OR: odds ratio
