# Supplementary material for: Transmission of Multidrug-Resistant and Drug-Susceptible Tuberculosis within Households: A Prospective Cohort Study
Source: PLoS Med. 2015 Jun 23;12(6):e1001843. doi: 10.1371/journal.pmed.1001843 (PMC4477882; doi:10.1371/journal.pmed.1001843)
Supplement: S1 Checklist — (DOCX) [file pmed.1001843.s001.docx]

STROBE Statement—checklist of items that should be included in reports of observational studies

|  | Item No. | Recommendation | Paragraph No. per Section | Relevant text from manuscript |
| --- | --- | --- | --- | --- |
| **Title and abstract** | 1 | (*a*) Indicate the study’s design with a commonly used term in the title or the abstract | Title Page  Paragraph 1 | Transmission of Multidrug-Resistant and Drug Susceptible Tuberculosis Within Households: A Prospective Cohort Study |
|  |  | (*b*) Provide in the abstract an informative and balanced summary of what was done and what was found | Title Page Paragraph 7 | Background  The 'fitness' of an infectious pathogen is defined as the ability of the pathogen to survive, reproduce, be transmitted and cause disease. The fitness of multidrug-resistant tuberculosis (MDRTB) relative to drug susceptible tuberculosis is cited as one of the most important determinants of MDRTB spread and epidemic size. To estimate the relative fitness of drug resistant tuberculosis cases, we compared the incidence of tuberculosis disease among the household contacts of MDRTB index patients to that among the contacts of drug susceptible index patients.  Methods and Findings  This 3-year (2010-2013) prospective cohort household follow-up study in South Lima and Callao, Peru, measured the incidence of tuberculosis disease among 1055 household contacts of 213 multidrug-resistant tuberculosis (MDRTB) index cases and 2362 household contacts of 487 drug susceptible index cases.  A total of 35/1055 (3·3%) household contacts of 213 MDRTB index cases developed tuberculosis disease, while 114/2362 (4·8%) household contacts of 487 drug susceptible index patients developed tuberculosis disease. The total follow-up time for drug susceptible tuberculosis contacts was 2620 years while the total follow-up time for multidrug-resistant tuberculosis contacts was 1425 years. Using multivariate Cox regression to adjust for confounding variables including contact HIV status, contact age, socio-economic status and index sputum smear grade, the Hazard Ratio (HR) for tuberculosis disease among MDRTB household contacts was found to be half that for drug susceptible contacts (HR 0·56, 95% CI 0·34-0·90, p=0·017). The inference of transmission in this study was limited by the lack of genotyping data for household contacts. Only capturing incident household disease may also limit the extrapolation of these findings to the community setting.  Conclusions  The low relative fitness of MDRTB estimated by this study improves the chances of controlling drug resistant tuberculosis. However, fitter multidrug-resistant strains that emerge over time may make this increasingly difficult. |
| Introduction | | | |  |
| Background/rationale | 2 | Explain the scientific background and rationale for the investigation being reported | Introduction Paragraph 2 | Studies by Mitchison [6] and Middlebrook [7] established in animal models that some drug resistant strains of tuberculosis were less pathogenic. Population level molecular epidemiological studies support this finding. These studies estimate tuberculosis fitness by measuring the proportion of strains that are genetically clustered and attributable to recent transmission [8–10]. More recently laboratory competitive fitness assays have demonstrated a variable fitness cost of drug resistant *M. tuberculosis* bacilli with most strains demonstrating a fitness cost and some with superior fitness [11–13]. However studies of this kind do not account for the myriad of potential clinical, environmental and socio-economic confounding variables that influence the ability of a patient to transmit the pathogen and cause tuberculosis disease in a contact. *In vitro* techniques also fail to measure fitness over the transmission cycle of the pathogen from disease in the index case to disease in the contact.  Very few studies have estimated fitness *in vivo* by comparing the incidence of second cases of tuberculosis among contacts of multidrug-resistant tuberculosis patients to that among the contacts of drug susceptible tuberculosis patients. Studies that have measured the incidence of second cases in households with multidrug-resistant tuberculosis have lacked statistical power [11,12], or have not included drug susceptible controls for comparison. [13] |
| Objectives | 3 | State specific objectives, including any prespecified hypotheses | Methods Paragraph 2 | . . . . hypothesis was that the incidence of second cases of tuberculosis disease among the contacts of MDRTB was different to that among the contacts of drug susceptible tuberculosis contacts. |
| Methods | | |  | |
| Study design | 4 | Present key elements of study design early in the paper | Methods Paragraph 2 | This 3 year prospective cohort study |
| Setting | 5 | Describe the setting, locations, and relevant dates, including periods of recruitment, exposure, follow-up, and data collection | Methods Paragraph 2 | This 3 year prospective cohort study was undertaken between September 2010 and September 2013 in two study sites in metropolitan Lima (Lima South and the region of Callao). |
| Participants | 6 | (*a*) *Cohort study*—Give the eligibility criteria, and the sources and methods of selection of participants. Describe methods of follow-up  *Case-control study*—Give the eligibility criteria, and the sources and methods of case ascertainment and control selection. Give the rationale for the choice of cases and controls  *Cross-sectional study*—Give the eligibility criteria, and the sources and methods of selection of participants | Methods Paragraph 3 | Multidrug resistant tuberculosis patients (resistant to at least rifampicin and isoniazid) and drug susceptible patients (susceptible to both rifampicin and isoniazid) were identified at diagnosis from each of the regional reference laboratories. Index patients were recruited at diagnosis (MDR or drug susceptible) and a sputum culture was obtained. |
|  |  | (*b*) *Cohort study*—For matched studies, give matching criteria and number of exposed and unexposed  *Case-control study*—For matched studies, give matching criteria and the number of controls per case | Methods Paragraph 4 | Each MDRTB index patient recruited at least two age and sex matched drug susceptible controls were selected at random from the same study site as the index case for comparison. |
| Variables | 7 | Clearly define all outcomes, exposures, predictors, potential confounders, and effect modifiers. Give diagnostic criteria, if applicable | Methods Paragraph 5-6 | Index patients were followed up at the local health centre by study staff every 6 months and asked about the wellbeing of family members and the occurrence of second cases of tuberculosis disease in the household. Any symptomatic contacts were encouraged to attend the local health post for testing. When recruitment to the study ended in January 2013, all index patients and their families were visited at home to ensure that all incident cases of tuberculosis disease during the study period had been recorded. The final round of active household based follow-up for all families was designed to minimize potential bias from variable follow-up between groups. Chemoprophylaxis was prescribed and managed by the treating health centre/physician in accordance with the national tuberculosis policy: National policy recommended chemoprophylaxis to the household contacts of drug susceptible tuberculosis <16 years of age and not for the contacts of MDRTB household contacts [19]. When there were delays in confirming MDRTB in the index case, multidrug-resistant tuberculosis contacts below 16 years of age were given isoniazid chemoprophylaxis, however after confirming the diagnosis the chemoprophylaxis was stopped. The number of contacts taking chemoprophylaxis and the duration of chemoprophylaxis was recorded for both groups.    Household contacts were defined as any person living in the same house as the index case for more than one day a week. Follow-up time started for MDRTB household contacts at the time of diagnosis of index case MDRTB, while follow up time for drug susceptible index patients and their contacts started at the time of drug susceptible tuberculosis diagnosis. An ‘event’ was defined as the development of tuberculosis disease in a household contact that occurred after the diagnosis of tuberculosis in the index case. Tuberculosis disease in household contacts was defined as any patient that had evidence of tuberculosis disease from sputum smear, culture, chest x-ray or clinical diagnosis that led to initiation of anti-tuberculous treatment. This definition was chosen in order to include children with tuberculosis who were diagnosed with tuberculosis disease and started on anti-tuberculous treatment by the treating physician without a microbiologically confirmed diagnosis. Contacts were censured (follow up stopped) if they were lost to follow-up or died. The incidence of tuberculosis disease was calculated as the number of incident cases of tuberculosis divided by the total number of contact follow-up person-years. Odds ratios of the differences between the two comparison groups were calculated using the STATA csi command for odds ratios in cohort studies with an exact p-value. Previous tuberculosis disease was defined as any disease episode in which anti-tuberculous treatment had been successfully completed more than 6 months prior to the onset of the present tuberculosis episode. |
| Data sources/ measurement | 8* | For each variable of interest, give sources of data and details of methods of assessment (measurement). Describe comparability of assessment methods if there is more than one group | Methods Paragraph 4 | The structured questionnaire recorded information on patient demographics, the household environment (crowding and house construction) and clinical data such as sputum smear result, culture results and diagnosis dates. All variables had been designed and field tested during the preliminary retrospective study [18].  All tuberculosis patients in Peru are tested for human immunodeficiency virus (HIV); these data were available from the patient record.  Patients that were able to expectorate sputum had their sputum tested by serial smear microscopy on a monthly basis. For the purposes of analyzing time to sputum conversion to negative during index case follow-up; index patients were regarded as becoming smear negative on the date of the first negative sample if they consistently remained smear negative thereafter. Index patients were also regarded as being smear negative if they could no longer produce sputum and continued as such.  Socio Economic Status was derived from the Necesidades Basicas Insatisfechas score, a locally validated scoring system used as part of the Peruvian National Census. This allows distinction to be made between different levels of poverty within a shanty town. |
| Bias | 9 | Describe any efforts to address potential sources of bias | Methods Paragraph 4 | In order to minimize bias, for each MDRTB index patient recruited at least two age and sex matched drug susceptible controls were selected at random from the same study site as the index case for comparison.  The final round of active household based follow-up for all families was designed to minimize potential bias from variable follow-up between groups. |
| Study size | 10 | Explain how the study size was arrived at | Methods Paragraph 2 | Incidence rates of tuberculosis disease from previous studies [17,18] were used to perform a power calculation to determine the sample size for a detectable alternative hazard ratio with a power of 0.8. A minimum MDRTB contact sample size of 800 with 1600 drug susceptible contacts (a 2:1 ratio) was determined to yield a significant difference between the two groups (two-tailed p<0.05) with a hazard ratio ≤0.6. |

Continued on next page

| Quantitative variables | 11 | Explain how quantitative variables were handled in the analyses. If applicable, describe which groupings were chosen and why | Methods Paragraph 4  And Table 1 | Socio Economic Status was derived from the Necesidades Basicas Insatisfechas score, a locally validated scoring system used as part of the Peruvian National Census. This allows distinction to be made between different levels of poverty within a shanty town.  All variables had been designed and field tested during the preliminary retrospective study [18]. |
| --- | --- | --- | --- | --- |
| Statistical methods | 12 | (*a*) Describe all statistical methods, including those used to control for confounding | Methods Paragraph 10 | Independent predictors of second cases were determined using a multivariate Cox regression survival analysis clustered at the level of the household. Each variable was tested for potential violation of the proportional hazards assumption by minus log-log plots and examination of the Schoenfeld residuals. Correction for clustering was undertaken at the level of the household to account for the correlation of variance within households. The clustering method used was to employ the STATA cluster clustvar command to provide a robust estimate of the standard error according to the Huber/White/Sandwich estimate of variance [23]. Known confounding variables identified a-priori (specifically HIV status of contacts, contact age, contact sex, sputum smear status of the index case and socio-economic status) together with variables which were found to be p<0·2 on univariate analysis were included in the multivariate regression and biologically plausible interactions were also examined for significance. A p-value of <0·05 was considered statistically significant in the multivariate regression. Analysis was undertaken using Stata (StataCorp. 2009. *Stata Statistical Software: Release 11*. College Station, TX: StataCorp LP). The pre-planned analysis did not differ from the final analysis other than consideration of the potentially confounding association of index case genotype and second cases of tuberculosis disease which was undertaken after reviewers comments. |
|  |  | (*b*) Describe any methods used to examine subgroups and interactions | Methods Paragraph 10 | The interactions between age and chemotherapy use, diabetes and index drug resistance, socio-economic status and sputum smear grade, contact employment and socio-economic status as well as index case genotype and index case drug resistance status were all examined for significance in predicting second household cases of disease. |
|  |  | (*c*) Explain how missing data were addressed | Methods Paragraph 8 | Missing data was minimized by revisiting household and health centres and cross checking interview data against medical records. Missing values were then treated as "missing at random" using Stata's default "listwise" deletion when included in multivariate regression analysis. |
|  |  | (*d*) *Cohort study*—If applicable, explain how loss to follow-up was addressed  *Case-control study*—If applicable, explain how matching of cases and controls was addressed  *Cross-sectional study*—If applicable, describe analytical methods taking account of sampling strategy | Methods Paragraph 8 | Contacts were censured (follow up stopped) if they were lost to follow-up or died. |
|  |  | (*e*) Describe any sensitivity analyses | N/A | N/A |
| Results | | | | |
| Participants | 13* | (a) Report numbers of individuals at each stage of study—eg numbers potentially eligible, examined for eligibility, confirmed eligible, included in the study, completing follow-up, and analysed | Fig. 1 | This has been given in the flow diagram |
|  |  | (b) Give reasons for non-participation at each stage | Results Paragraph 1 | . . . could not be recruited - forty five patients (48% of un-recruited patients) could not be located either at the health post or at home as an erroneous address had been provided or they had abandoned treatment at the health post after having left a diagnostic specimen, 20 (22%) had died before an interview could be undertaken, 16 (17%) were imprisoned and 12 (13%) chose not to consent to enter the study. This left the household contacts of 213 newly diagnosed MDRTB index cases that were followed up as part of the study. |
|  |  | (c) Consider use of a flow diagram | Fig. 1 | See flow diagram |
| Descriptive data | 14* | (a) Give characteristics of study participants (eg demographic, clinical, social) and information on exposures and potential confounders | Table 1 and Table 2 | See Table 1 and 2 |
|  |  | (b) Indicate number of participants with missing data for each variable of interest | Table 1 and Table 2 | See Table 1 and 2 |
|  |  | (c) *Cohort study*—Summarise follow-up time (eg, average and total amount) | Results Paragraph 6 | The total follow up time of MDRTB contacts was 1425 years (mean follow up time per MDRTB contact 494 days, standard deviation 199 days) in which 35 second cases arose, equating to an incidence of 2456 per 100,000 contact follow up years. The total follow up time of drug susceptible contacts was 2620 (mean follow up time per drug susceptible contact 406 days, standard deviation 189 days) years in which 114 second cases arose, equating to an incidence of 4351 per 100,000 contact follow up years (HR 0·56 95% CI 0·34-0·90, p=0·017, Fig. 2, multivariate analysis). |
| Outcome data | 15* | *Cohort study*—Report numbers of outcome events or summary measures over time | Fig.1 to 5 | See Figure 1 to 5 which present outcome events over study time. |
|  |  | *Case-control study—*Report numbers in each exposure category, or summary measures of exposure |  | N/A |
|  |  | *Cross-sectional study—*Report numbers of outcome events or summary measures |  | N/A |
| Main results | 16 | (*a*) Give unadjusted estimates and, if applicable, confounder-adjusted estimates and their precision (eg, 95% confidence interval). Make clear which confounders were adjusted for and why they were included | Table 3,4,5. | See Table 3, 4 and 5. |
|  |  | (*b*) Report category boundaries when continuous variables were categorized | Table 2,3,4,5 | See Table 2,3,4 and 5. |
|  |  | (*c*) If relevant, consider translating estimates of relative risk into absolute risk for a meaningful time period |  | N/A |

Continued on next page

| Other analyses | 17 | Report other analyses done—eg analyses of subgroups and interactions, and sensitivity analyses | Methods Paragraph 10 | The interactions between age and chemotherapy use, diabetes and index drug resistance, socio-economic status and sputum smear grade, contact employment and socio-economic status as well as index case genotype and index case drug resistance status were all examined for significance in predicting second household cases of disease. |
| --- | --- | --- | --- | --- |
| Discussion | | | | |
| Key results | 18 | Summarise key results with reference to study objectives | Discussion Paragraph 1 | This prospective cohort study has demonstrated that over 3 years follow up the incidence of tuberculosis disease in households with an index MDRTB case is almost half that of households with a drug susceptible index case. |
| Limitations | 19 | Discuss limitations of the study, taking into account sources of potential bias or imprecision. Discuss both direction and magnitude of any potential bias | Discussion Paragraph 7 | Our study has a number of important strengths and limitations. A large cohort of MDRTB patients followed over 3 years, the highest risk period for incident tuberculosis disease following acquisition of new infection, enabled us to recruit enough newly diagnosed patients to accurately compare the incidence of disease in both groups with sufficient statistical power to detect a difference between the two groups. Comprehensive index patient interviews gave us detailed data on potentially confounding clinical, demographic and socio-economic variables, while active case finding visits to the household maximized the sensitivity of case detection. Genotyping culture positive contacts would have allowed us to be more certain of the relative contribution of extra-domiciliary transmission to MDRTB and drug susceptible households. [27] However, drug susceptible control patients were selected from the same region as drug resistant index cases and were therefore likely to have been exposed to a similar risk of tuberculosis infection from the surrounding community. |
| Interpretation | 20 | Give a cautious overall interpretation of results considering objectives, limitations, multiplicity of analyses, results from similar studies, and other relevant evidence | Discussion Paragraph 5 | The incidence rate of disease among MDRTB contacts in this study was almost identical to our previous estimate of the incidence of disease in MDRTB contacts established in a preliminary retrospective study [24] and the disease yield among drug susceptible contacts in this study is very similar to those reported elsewhere [18].  The most recently published survey by the World Health Organization [20] in October 2014 supports our findings demonstrating that globally the proportion of new cases of MDRTB has not changed between 2008-2013 remaining at 3.5% of new cases. |
| Generalisability | 21 | Discuss the generalisability (external validity) of the study results | Discussion Paragraph 4 | The fitness of MDRTB estimated here must also be taken in the context of the national tuberculosis control program and the household follow-up study design. This may limit the extrapolation of these findings to other countries, particularly when considering outbreaks in the community or prisons where conditions may favour the spread of MDRTB. Contacts outside the house may come into contact with and be infected by the index case at any stage during the infectious period (which because of delays in diagnosis is longer for MDRTB patients), while contacts inside the house because of frequent exposure are more likely to be infected earlier in the infectious period. This factor could increase the number of second cases and hence MDRTB fitness estimates in the community. |
| Other information | |  | | |
| Funding | 22 | Give the source of funding and the role of the funders for the present study and, if applicable, for the original study on which the present article is based | Online documentation | Funding was provided by the Wellcome Trust, grant number WT088559MA (www.wellcome.ac.uk). The funders had no role in study design, data collection and analysis, decision to publish, or preparation of the manuscript. |

*Give information separately for cases and controls in case-control studies and, if applicable, for exposed and unexposed groups in cohort and cross-sectional studies.

**Note:** An Explanation and Elaboration article discusses each checklist item and gives methodological background and published examples of transparent reporting. The STROBE checklist is best used in conjunction with this article (freely available on the Web sites of PLoS Medicine at http://www.plosmedicine.org/, Annals of Internal Medicine at http://www.annals.org/, and Epidemiology at http://www.epidem.com/). Information on the STROBE Initiative is available at www.strobe-statement.org.
