# Supplementary material for: Transmission of Multidrug-Resistant and Drug-Susceptible Tuberculosis within Households: A Prospective Cohort Study
Source: PLoS Med. 2015 Jun 23;12(6):e1001843. doi: 10.1371/journal.pmed.1001843 (PMC4477882; doi:10.1371/journal.pmed.1001843)
Supplement: S1 Text — (PDF) [file pmed.1001843.s002.pdf]

UBI VULT SPIRITUS SPIRAT
UNIVERSIDAD PERUANA CAYETANO HEREDIA

Vicerrectorado de Investigación
Dirección Universitaria de Investigación, Ciencia y Tecnología - DUICT

CONSTANCIA

El que suscribe, Presidente del Comité Institucional de Ética (CIE) de la Universidad Peruana Cayetano Heredia certifica que ha reevaluado el Proyecto de Investigación titulado: "**Tuberculosis activa en contactos de los pacientes con Tuberculosis MDR-Un estudio Prospectivo"**, aprobado inicialmente en fecha 29 de noviembre del 2010, código de inscripción **57492**, presentado por el Investigador Principal, **Dr. Louis Grandjean.**

Considerando que:

1. El protocolo de investigación mantiene los lineamientos **científicos y éticos**, con balance favorable entre **riesgos y beneficios.**
2. El equipo investigador se encuentra calificado para su ejecución
3. El **Consentimiento Informado y Asentimiento Informado** brindan la información necesaria de manera adecuada y permiten el enrolamiento voluntario de los participantes.
4. Están contemplados mecanismos de **confidencialidad** de los datos clínicos.

De acuerdo a los estándares propuestos por la Universidad.

Por lo tanto, el CIE acuerda **EXPEDIR LA REAPROBACION**, en fecha **22 de noviembre del 2011** por seguir las Guías de Buenas Prácticas Clínicas.

Documentos incluidos en esta reaprobación:

- **Protocolo de investigación**, versión Final de Fecha 23 de octubre de 2010, aprobado por el CIE en sesión de fecha 29 de noviembre de 2010.
- **Consentimiento Informado, vigilancia activa: adultos,** versión final 26 de noviembre de 2010, aprobado por el CIE en sesión de fecha 29 de noviembre de 2010**.**
- **Consentimiento Informado para Padres,** versión final 26 de noviembre de 2010, aprobado por el CIE en sesión de fecha 29 de noviembre de 2010.

…///

Av. Honorio Delgado 430, lima 31, Perú. Apartado Postal 4314, Lima 100
Teléfono: (511) 319-0000 Anexo: 2271 / 2542 **Telefax:** 482-4541
E-mail: duict@oficinas-upch.pe
Página Web: http://www.upch.edu.pe/vrinve/duict/

UBI VULT SPIRITUS SPIRAT
UNIVERSIDAD PERUANA CAYETANO HEREDIA
Vicerrectorado de Investigación
Dirección Universitaria de Investigación,
Ciencia y Tecnología - DUICT

////.......

- **Asentimiento Informado, vigilancia pasiva: Niños y Adolescentes 9 a 17 años,** versión final 26 de noviembre de 2010, aprobado por el CIE en sesión de fecha 29 de noviembre de 2010.

Cualquier **enmienda** al protocolo original, así como la ocurrencia de alguna **eventualidad** que afecte adversamente a los participantes, deberá ser reportada en breve plazo a este Comité.

El investigador reportará cada 6 meses el progreso del estudio y alcanzará un **informe** al término de éste.

Esta reaprobación tendrá **vigencia hasta el 21 de noviembre del 2012**. Los trámites para su renovación deberán iniciarse por lo menos 30 días previos a su vencimiento.
El investigador presentará un informe al término del estudio

Lima, 22 de noviembre de 2011

Dr. Leonid Lecca García
Presidente
Comité Institucional de Ética
/zrmg

COMITÉ INSTITUCIONAL DE ÉTICA - CIE
UNIVERSIDAD PERUANA CAYETANO HEREDIA
DUICT

Av. Honorio Delgado 430, lima 31, Perú. Apartado Postal 4314, Lima 100
Teléfono: (511) 319-0000 Anexo: 2271 / 2542 **Telefax:** 482-4541
E-mail: duict@oficinas-upch.pe
Página Web: http://www.upch.edu.pe/vrinve/duict/

UNIVERSIDAD PERUANA
CAYETANO HEREDIA

Vicerrectorado de Investigación
Dirección Universitaria de Investigación,
Ciencia y Tecnología - DUICT

Lima, 22 de Noviembre de 2011

Doctor
LOUIS GRANDJEAN
Investigador principal
Presente.-

Ref.: Proyecto 57492:" Tuberculosis activa en contactos de los pacientes con Tuberculosis MDR-Un estudio Prospectivo"

Estimado Dr. Grandjean:

El que suscribe, Presidente del Comité Institucional de Ética (CIE) de la Universidad Peruana Cayetano Heredia, certifica que en reunión del Comité Institucional de Ética, de fecha 22 de Noviembre del 2011, **SE TOMO CONOCIMIENTO de:**

- ✓ Informe de Avances del Estudio de la referencia

Como nos informara en los documentos recibidos en fecha 15 de noviembre de 2011.

Atentamente,

COMITÉ INSTITUCIONAL DE ÉTICA - CIE
UNIVERSIDAD PERUANA CAYETANO HEREDIA DUICT

Dr. Leonid Lecca García
Presidente
Comité Institucional de Ética.

/zrmg

Av. Honorio Delgado 430, lima 31, Perú. Apartado Postal 4314, Lima 100
Teléfono: (511) 319-0000 Anexo: 2271 / 2542 **Telefax:** 482-4541
E-mail: duict@oficinas-upch.pe
Página Web: http://www.upch.edu.pe/vrinve/duict/

Tuberculosis Disease Among the Contacts of Drug Resistant Tuberculosis - A Prospective Study

Study Description

The treatment of multidrug-resistant tuberculosis (MDRTB) and extensively drug resistant tuberculosis (XDRTB) requires a combination of second line anti-tuberculous agents that in general are less efficacious and more toxic than first line therapies[1]. Resistance to isoniazid in the index case with active disease renders isoniazid prophylactic chemotherapy to latently infected contacts ineffective. The potential utility of second line agents to prevent active disease among latently infected contacts of multidrug-resistant tuberculosis has not been evaluated[2]. Before considering the use of second line drug to prevent MDRTB and XDRTB it is necessary to: a) establish the incidence of tuberculosis among the contacts of drug susceptible and drug resistant household contacts to define the maximum potentially avoidable morbidity b) to determine the concordance of the drug resistance profile between the index case and the contact and therefore c) the number needed to treat (NNT) to prevent one case of resistant tuberculosis. This prospective cohort study will establish the incidence of second cases of disease among the household contacts of MDRTB and drug susceptible index cases. It will also establish the extent of concordance of drug susceptibility test between the index case and the diseased household contact and the factors associated with concordance. This study will also evaluate whether there is a potential to use second line anti-tuberculous drug to prevent the development of MDRTB among household contacts. The study will begin in October 2010 in the regions of Lima South and Callao. Households with either a drug susceptible index case, or an MDRTB index case diagnosed by the regional reference laboratory will be visited. Each index case will be asked to help complete a questionnaire in which we will record demographic, clinical and environmental data. This data will be cross referenced with data recorded in the medical records and data compiled by the national institute of health and technical MDRTB unit. Households will be visited every 3 months to evaluate the health of the index case and that of the household contacts. In collaboration with the technical MDRTB unit and the Peruvian Ministry of Health, the index case sputum samples and those of unwell contacts will be processed by the regional reference laboratory then divided to store half the sample at the Universidad Peruana Cayetano Heredia for genotyping. Data will be securely stored and analyzed using STATA 10 software.

Objective of the Study

The objective of the study is to estimate the incidence of active tuberculosis disease among the household contacts of MDRTB and drug susceptible tuberculosis. The study will also determine the extent and predictors of phenotypic concordance between the index case and the diseased household contact.

Objectives

1. To determine the incidence of second cases of disease among the household contacts of MDRTB and drug susceptible tuberculosis.

2. To determine the frequency of concordance between the index case and second case phenotype and genotype.

3. To determine the risk factors (medical, environmental and clinical) associated with the development of second cases of disease among household contacts.

Background

Isoniazid for the control and prevention of latent tuberculosis infections (LTBI)

Tuberculosis control requires that index cases with tuberculosis disease are treated, and when resources allow, that close contacts with latent tuberculosis infection are given isoniazid chemoprophylaxis to prevent the development of tuberculosis disease. Smeija et al[3] suggest that to prevent one case of tuberculosis over 5 years it is necessary to treat 35 contacts of the diseased index case. However because of the high cure rate of drug susceptible tuberculosis disease (85%)[4], the emphasis has been placed on first treating active tuberculosis disease. Therefore where resources are scarce, preventive chemotherapy is often considered an unnecessary use of these resources.

Preventive chemotherapy for the contacts of MDRTB

Treatment of multidrug-resistant tuberculosis (MDRTB) and extensively drug resistant tuberculosis (XDRTB) requires second line anti-tuberculous drugs which are less efficacious and more toxic than first line therapy[1]. Isoniazid resistance in MDRTB and XDRTB makes isoniazid ineffective in treating contacts latently infected with MDRTB. However the potential utility of second line agents for chemoprophylaxis of MDRTB is not clear. By definition it is not possible to determine with any certainty the drug susceptibility profile of contacts latently infected with drug-resistant tuberculosis therefore agents will need to be selected empirically based on the drug susceptibility profile of the index case. However, the case for preventing a case of drug resistant tuberculosis is made particularly strong given the relatively poor cure rates, the cost and toxicity associated with treating a case of MDRTB[1,5-6].

Methods

Study Design

Prospective Cohort Study

Location

Regions of Lima South and Callao, Peru

General Methods

Drug susceptible and MDRTB index cases will be identified at the regional reference laboratory in Callao and Lima South. Over the next 3 years patients who provide informed consent will be visited in their homes every 3 months to determine the existence of new household cases of tuberculosis in the home and the risk factors associated with second cases of tuberculosis disease. Rather than

discard the index case or diseased contact sputum sample, the regional reference laboratory will provide the Universidad Peruana Cayetano Heredia with an aliquot to be genotyped and stored in the Laboratory of Investigation and Development.

Phase 1

Recruiting the Index Cases

Drug susceptible index cases (1500 controls) and MDRTB index cases (650) will be identified when they are diagnosed by the regional reference laboratory of Callao and Lima South.

Phase 2

Household visits

Each person identified in phase 1 will be asked to provide informed consent then will be visited in their household and asked to help complete a questionnaire recording clinical, demographic and environmental[7] data. Over the next 5 years index cases and their contacts will be visited in their households every 3 months to identify new household cases of tuberculosis disease.

Health Post Visits

The health post of each index cases and diseased contact will also be visited to verify data, confirm drug susceptibility data, demographic and clinical details.

Phase 3

Multivariate Cox regression will be used to establish the demographic, environmental and clinical factors associated with second cases of tuberculosis disease.

Study Population

Two cohorts (MDRTB index cases and drug susceptible controls) will both be selected from the same region at the same time and be microbiologically confirmed. All the household contacts present in the house from the moment of diagnosis will be included in the study.

Evidence from Previous Studies

Previous studies of MDRTB contacts make it possible to estimate the number of probable household contacts with infection and disease (Table 2). The rate of transmission of tuberculosis in these studies varies depending on many factors including study site, contact age and HIV prevalence of the population.

Based on these previous studies we estimate that 650 drug susceptible index cases will generate on average 3250 household contacts. Of these 3250 contacts approximately 1170 (36% or 12% per year over 5 years)* will be infected with latent tuberculosis within the 5 years after the index case

*This preliminary power calculation was superseded by a more detailed power calculation; part of the "S2 Text" document that took account of incidence, variable follow up time, a 2:1 ratio of drug susceptible to MDRTB contacts and a study duration of 3 years.

diagnosis. From the 3250 contacts it is estimated that 358 (11% or 3.6% per year during the first 3 years) will have developed tuberculosis disease within the 5 years after the index case diagnosis.

Table 2. Results of 9 studies[8-16] provide the data on which the rate of infection and disease among contacts is based.

Study	Index Cases	Number of Contacts	TST positive (infection in contacts)	Contacts with tuberculosis disease (number)	Percent of contacts with disease	Number of cultures available for analysis	Study Duration
Grandjean (Peru) 2008	358	2112	-	108	5.1%	90%	3 years
Bayona (Perú) 2003	192	945	-	72	8%	42 (4%)	4-5 years
Schaaf 2002 (Africana Sud)	73	125[a]	51%	29	23%	4 (3%)	30 months
Riechler[c] (EE.UU.) 2002	349	2095	32%	24	1%	-	Retrospective
Noertjojo[c] (Hong Kong) 2002	970	2678	-	41	2%	-	Retrospective
Teixeira (Brazil) 2001	26	133	44%	6	5%	6 (5%)	4 years
Beyer Y Schaaf 1995 (Africana Sud)	80	664 (171[b])	14%	52	34%	9 (6%)	1 year
Kritski 1996 (Brazil)	64	218	93%	17	8%	12 (6%)	4 years
Snider 1985 (EE.UU)[c]	180	601	40%	-	-		Retrospective
Siminel 1979 Rumania	931	3189	60%	No fiable	4%	-	-
TOTAL	1934	7459	-	241	-	-	-
MEDIO	242	932	36%	34%	11%	5%	-

[a]All contacts less than 5 years of age and only 116 contacts were included in the study.

[b]All contacts less than 5 years of age and only 155 were followed for the duration of the study.

[c]non-MDR Study

Selection of Subjects and Definitions

Regional laboratories of Callao and Lima South are already employing MODS (Microscopic Observation Drug Susceptibility) testing to identify MDRTB. MODS was developed by the laboratory in which the study will be performed. Therefore MDRTB cases and drug susceptible cases will be easily identified. After processing the sample, instead of discarding the sample, an aliquot of the sample will be provided for storage and genotyping at Universidad Peruana Cayetano Heredia.

Index Case Definition - All index cases will have drug susceptibility status confirmed microbiologically. Index cases are heterogeneous group that includes both primary and secondary drug resistance. The questionnaire is designed to delineate these groups to help define the study outcomes.

Household Contacts - Any person of any age that lives in the house at the moment of the diagnosis of the index case. If a contact that lived in the house leaves the home the date that they leave the home and their clinical status on leaving the home will be recorded. If the household contact dies during the follow up period the cause of death and the date of death will be recorded.

Second Cases - Any household contact that develops tuberculosis disease after that of the index case. The term second case will be used to include the possibility of tuberculosis infection occurring from outside the home or tuberculosis disease that has arisen from reactivation of previous latent infection.

Follow Up Time of the Contacts - Follow up time will be defined as the time from diagnosis of the MDRTB index case until the last household interview or until the date of tuberculosis diagnosis in the household contact. However if the contact leaves the house or dies during the follow up period this date will be recorded and they will be regarded lost to follow up.

Method of Data Collection

Every home will be visited in person by the study staff, whenever possible in coordination with the health post and the personnel of the ESPCT. The head of the family will be asked to provide informed consent if they wish to participate in the study. Once they have agreed to join the study they will be asked to complete a structured questionnaire that includes demographic, clinical and environmental variables.

Data Management and Statistical Analysis

All the study data will be stored in the secure offices of the laboratory of investigation and development at Universidad Peruana Cayetano Heredia. Laboratory codes will be used instead of patient names to prevent a breach of confidentiality. Digital data will be stored on a password protected laptop dedicated to the study. The principle outcome measures of interest include:

1) The incidence of tuberculosis disease among the household contacts of MDRTB and drug susceptible index cases.

2) The proportion of second cases phenotypically and genotypically concordant with the index case.

3) The risk factors associated with the development of a second case of tuberculosis in the house.

Potential Benefits and Applications of the Study

The findings of this study will provide data that will allow health systems to understand the incidence of disease among the household contacts of multidrug resistant tuberculosis patients. It will also establish the demographic, clinical and environmental risk factors for a second case occuring. The study will provide the basis for the future evaluation and utilization of second line drugs for the prevention of MDRTB. The Ministry of Health will be informed of any new subject identified as part of the study this will permit treatment to begin immediately in accordance with the national guidelines.

Time Line

2010	2011	2012	2013	2014	2015
Data Collection October 2010	Data Collection and sample processing	Data Collection and sample processing	Data Collection and sample processing	Data Collection and sample processing	Publication of Results

Strengths and Limitations of the Study

The Lima suburbs have a high incidence of MDRTB and are therefore an ideal place to undertake the study. Study investigators have collaborated with the technical MDRTB unit of the Peruvian Ministry of Health in a similar study in 2006-7. The study will allow health systems to better understand the burden of second cases of disease in MDRTB households. Before evaluation of second line drugs for the prevention of MDRTB can be evaluated first the burden of disease in MDRTB household and the phenotypic concordance between index and contact must be understood.

This is a prospective study that will provide more reliable data collection that retrospective studies which are liable to recall bias. All data will be cross-referenced with medical information at the local health post.

Ethical Considerations

Ethical approval for the project will be obtained from the Universidad Peruana Cayetano Heredia and institutional approval will be obtained from the DISA de Callao and the DISA Lima South as well as the Peruvian Ministry of Health before the study begins. Every household index case will provide informed consent before they agree to participate in the study. Information on participants will be recorded in the questionnaire and cross-referenced with health post data and that from the reference laboratory. Whenever possible the laboratory will use codes instead of names to ensure patient confidentiality. Data will be stored securely in the offices of the Universidad Peruana Cayetano Hereida in the Laboratory of Investigation and Development (LID).

Local Collaborators

- Dr. Louis Grandjean and Dr. David Moore, Laboratorio de Investigación de Enfermedades Infecciosas, Universidad Peruana Cayetano Heredia (UPCH) and London School of Hygeine and Tropical Medicine.
- Dra Valentina Alarcon, Unidad Tecnica para Tuberculosis Multidrogoresistente sp, Ministerio de Salud
- Blga. Edith Castillo Acevedo, Laboratorio Regional de Micobacteriologia, DIRESA de Callao

References

1. Cox HS, Kalon S, Allamuratova S, et al. Multidrug-Resistant Tuberculosis Treatment Outcomes in Karakalpakstan, Uzbekistan: Treatment Complexity and XDR-TB among Treatment Failures. PLoS ONE 2007;2(11):e1126.

2. World Health Organisation. Guidelines for the Programmatic Management of Drug Resistant Tuberculosis. 2006.

3. Smieja MJ, Marchetti CA, Cook DJ, Smaill FM. Isoniazid for preventing tuberculosis in non-HIV infected persons. Cochrane Database Syst Rev 2000(2):CD001363.

4. World Health Organisation. Global Tuberculosis Control 2008: Surveillance, Planning, Financing. 2008.

5. World Health Organisation. Anti Tuberculosis Drug Resistance in the World 4th Report. 2008.

6. Becerra MC, Freeman J, Bayona J, et al. Using treatment failure under effective directly observed short-course chemotherapy programs to identify patients with multidrug-resistant tuberculosis. Int J Tuberc Lung Dis 2000;4(2):108-14.

7. Feres J, Mancero X. El método de las necesidades básicas insatisfechas (NBI) y sus aplicaciones en America Latina. In: 4to Taller regional del "Programa para el Mejoramiento de las Encuestas y la Medición de las Condiciones de Vida en América Latina y el Caribe" (MECOVI); 2001; Buenos Aires: United Nations; 2001.

8. Bayona J, Chavez-Pachas AM, Palacios E, Llaro K, Sapag R, Becerra MC. Contact investigations as a means of detection and timely treatment of persons with infectious multidrug-resistant tuberculosis. Int J Tuberc Lung Dis 2003;7(12 Suppl 3):S501-9.

9. Siminel M, Bungetzianu G, Anastasatu C. The Risk of Infection and Disease in Contacts with Patients Excreting Mycobacterium Tuberculosis Sensitive and Resistant to Isoniazid. Bulletin of the International Journal of Tuberculosis 1979.

10. Teixeira L, Perkins MD, Johnson JL, et al. Infection and disease among household contacts of patients with multidrug-resistant tuberculosis. Int J Tuberc Lung Dis 2001;5(4):321-8.

11. Snider DE, Jr., Kelly GD, Cauthen GM, Thompson NJ, Kilburn JO. Infection and disease among contacts of tuberculosis cases with drug-resistant and drug-susceptible bacilli. Am Rev Respir Dis 1985;132(1):125-32.

12. Schaaf HS, Gie RP, Kennedy M, Beyers N, Hesseling PB, Donald PR. Evaluation of young children in contact with adult multidrug-resistant pulmonary tuberculosis: a 30-month follow-up. Pediatrics 2002;109(5):765-71.

13. Reichler MR, Reves R, Bur S, et al. Evaluation of investigations conducted to detect and prevent transmission of tuberculosis. Jama 2002;287(8):991-5.

14. Kritski AL, Marques MJ, Rabahi MF, et al. Transmission of tuberculosis to close contacts of patients with multidrug-resistant tuberculosis. Am J Respir Crit Care Med 1996;153(1):331-5.

15. Beyers N, Gie RP, Schaaf HS, et al. A prospective evaluation of children under the age of 5 years living in the same household as adults with recently diagnosed pulmonary tuberculosis. Int J Tuberc Lung Dis 1997;1(1):38-43.

16. Noertjojo K, Tam CM, Chan SL, Tan J, Chan-Yeung M. Contact examination for tuberculosis in Hong Kong is useful. Int J Tuberc Lung Dis 2002;6(1):19-24.
