# Supplementary material for: Transmission of Multidrug-Resistant and Drug-Susceptible Tuberculosis within Households: A Prospective Cohort Study
Source: PLoS Med. 2015 Jun 23;12(6):e1001843. doi: 10.1371/journal.pmed.1001843 (PMC4477882; doi:10.1371/journal.pmed.1001843)
Supplement: S2 Text — (PDF) [file pmed.1001843.s003.pdf]

Chapter 6

Literature Review: Tuberculosis Household Follow-Up Studies

6.1 Tuberculosis Contact Follow-Up Studies

6.1.1 The Need to Study MDRTB Contacts

It is theoretically possible to prevent new cases of MDRTB using chemoprophylaxis selected on the basis of the index case strain ('tailored chemoprophylaxis') [145,148,332]. Before a clinical trial can evaluate the potential of 'tailored chemoprophylaxis' the extent to which the index case phenotype and genotype match that of subsequent household contacts must be determined. Without this knowledge there is a real risk of treating presumed latent MDRTB infection with inappropriate antibiotic therapy and a potential of generating new resistance to precious second line agents through inadequate therapy. The ability to predict genotypic and phenotypic concordance using clinical, environmental and strain data could allow tailored chemoprophylaxis to be more appropriately targeted to the groups in which it is most likely to have greatest impact and minimise iatrogenic drug resistance.

In order to predict the future course of drug resistant tuberculosis the extent to which MDRTB is transmitted in the community relative to that of drug susceptible tuberculosis must be understood. The relative transmission of MDRTB in the community provides a proxy measure of the transmissibility of the bacilli in its environment and is the critical component of predictive models of drug resistant tuberculosis [3,6].

Understanding the incidence of secondary tuberculosis disease in households with an MDRTB index case will allow public health program managers to more accurately allocate funds to control drug resistant tuberculosis. The cost implications of preventing

a case of MDRTB are enormous given that the cost of treating a patient with MDRTB is approximately 10 times that of a patient with drug susceptible tuberculosis [333,334].

6.1.2 Published Household Contact Follow Up Studies

Many studies have examined the outcome of the household contacts of patients with drug susceptible tuberculosis [286–303]. A recent meta-analysis of these studies [335] concluded that the pooled 'yield' of tuberculin skin test (TST) positive contacts was 51.4% (95% CI: 50.6–52.2) while the pooled 'yield' of active tuberculosis amongst household contacts was 4.5% (95% CI: 4.3%–4.8%). The heterogeneity between studies was significant (I^2=95.5%, P<0.01) and none of the studies considered the influence of variable contact follow-up time (incidence density rate i.e. incidence as measured per contact follow up year).

Only 8 studies to date have specifically examined tuberculosis infection and disease among household contacts of index patients with MDRTB [145,147,148,336–340]. The 'yields' of active tuberculosis disease reported vary from between 4% to 8% of contacts. Two of these studies (both conducted in Lima) did consider the incidence density, reporting an incidence density rate of 2928 per 100,000 contact follow up years (2.9%) and 2360 per 100,000 follow up years (2.4%) respectively [148,338].

The case control study by Snider et al [147] that measured the skin test conversion among household contacts of isoniazid and/or streptomycin resistant index cases and drug susceptible index cases has already been discussed in detail in section 2.5.2.

The study undertaken by Kritski et al [145] has also been discussed in the same section. Briefly this retrospective study followed up the HIV seronegative close contacts of 64 MDRTB (defined by the authors as resistance to any two drugs) index patients with culture confirmed disease. The study estimated that the yield of second cases of tuberculosis disease among MDRTB households was 7.8% (17/218) of household contacts. A total of 13 diseased household contacts had drug susceptibility tests performed, 6 of these (47%) had an identical phenotype to the index case, 31% were

discordant but also drug resistant and 23% were completely drug susceptible. Kritski et al concluded on the basis of these findings that one could not predict with certainty the phenotype of diseased household contacts.

Schaaf et al [336] followed up the paediatric contacts (less than 5 years old) of 73 multidrug-resistant tuberculosis cases in Cape Town. Over a 30 month follow up period 12% of children (14/119) were diagnosed with tuberculosis disease. Only 4 children had culture positive tuberculosis of which 3 had an identical genotype to that of the index case.

In 2003, Bayona et al [337] undertook a study in Lima, Peru that recruited 91 MDRTB index patients some of whom were chronically diseased and some of whom were new diagnoses of MDRTB. These index cases lived with a total of 945 household contacts of which 72 developed tuberculosis disease either around the time of the index case (termed a prevalent case) or after the index case (termed an incident case). The definition of a prevalent treated case included those patients who were found to be receiving treatment at the same time as enrolment of the index case. This therefore included patients diagnosed before the index case. The follow-up period was defined as the period from which the index case was enrolled until the end of the study. This definition therefore excludes a long period of time in which the index case may have been unwell and exposing the household contacts to drug resistant bacilli. This artificial shortening of the true exposure time may explain the extremely high yield (8.5%) seen in this study that has not been replicated elsewhere.

In 2011 Becerra et al [338] compared the incidence of disease in among household contact with an index XDRTB case to those households with an MDRTB index case. This study has also been discussed in section 2.5.2. In summary Becerra et al determined the incidence in case with XDRTB to exceed that of MDRTB.

In the same year Vella et al [339] published the results of a prospective study of the household contacts of MDRTB and XDRTB in Kwa-Zulu Natal which determined that the

incidence of disease among contacts was a minimum 1700/100,000. This study did not include children with tuberculosis disease, hence the authors stressed that this figure was likely to reflect a lower bound of incidence. In this study most second cases were diagnosed at the initial household visit and the vast majority within 6 months following the diagnosis of the index case.

A similar but retrospective study was undertaken at the same time by Grandjean et al [148] in Lima who estimated the incidence among household contacts of MDRTB index patients to be 2360/100,000. This study also demonstrated that contacts with a previous history of tuberculosis and non-HIV associated medical history had a higher incidence rate of disease.

6.1.3 Overview of Contact Study Methodology

The eight MDRTB contact 'follow up' studies published to date used a range of methodologies summarized in Table 1. Kritski et al. [145] defined MDRTB as any two drugs making the index case population heterogeneous. Most are retrospective studies which are inherently less suited to following up cohorts of patients. Hence there exists a real need to conduct a rigorous prospective cohort study of the contacts of MDRTB index patients.

Table 21 - A review of the methods used in the other studies of MDRTB contacts to date.

Study	Study Type	MDRTB Index Case Definition	Follow Up Technique	Definition of a Household Contact
**Siminel 1979**	Retrospective	None Given (Only a published abstract)	None Given (Only a published abstract)	None Given (Only a published abstract)
**Snider 1985 [147]**	Cross Sectional	Resistance to SM and/or INH Excluded Index Patients with Prior Therapy	Passive surveillance (sent a questionnaire to the health post)	Contacts below 15 years old ('high risk')
**Kritski 1996 [145]**	Prospective Cohort	Excluded Index Patients who had TB before MDRTB defined as two or more anti-tuberculous drugs	One Health Centre Interview of Index and Contacts at the outset	Living with Index ≥ 5 years
**Schaaf 2002 [336]**	Prospective	Resistance to RIF and INH	Heath post interview and follow up at the health post	Children <5 yrs living in the same house or group of clustered houses for > 1 month
**Teixeira 2001 [340]**	Prospective	Resistance to RIF and INH	Health Post Interviews	Shared the same house and kitchen as the index case for > 3 months before index started treatment
**Bayona 2003 [337]**	Retrospective	Resistance to RIF and INH	Household Interviews	Prolonged, frequent or intense contact with the individual when infectious
**Grandjean 2011 [148]**	Retrospective	Resistance to RIF and INH	Health Post and Household Follow Up	Living in the house of the index case during tuberculosis disease for more than 1 day per week
**Becerra 2011 [338]**	Retrospective	Resistance to RIF and INH	Health Post and Household Follow Up	Living with the index patient on the date that the MDR tuberculosis regimen was initiated
**Vella 2011 [339]**	Prospective	Resistance to RIF and INH	Household	>13 years old

6.1.4 Novelty of the Study

Although many studies have evaluated the yield of tuberculosis disease in households with drug susceptible tuberculosis [335], there have only been two prospective studies [339,340] and relatively few retrospective studies [148,332,337,338] that have examined the incidence of tuberculosis among the household contacts of MDRTB index patients. Only three retrospective studies have examined matching pairs of index-contact drug susceptibility tests [148,337,338] and a fourth study in children where only 5 pairs of index-contact DST were reported [332].

The WHO recommendations for chemoprophylaxis in household contacts of drug resistant tuberculosis are ambiguous, predominantly because of the lack of data regarding the likely utility of 'tailored chemoprophylaxis' [15]. This study will identify the concordance of index and contact drug susceptibility tests in MDRTB households and will therefore help rationalize the advice given to contacts of MDRTB. To date, no studies have examined the factors that influence index-contact concordance in MDRTB.

Chapter 7

Planning and Background to the Household Follow Up Study

7.1 Power Calculations

Based on previous studies of tuberculosis disease among the contacts of drug susceptible tuberculosis, three incidence density rates were used to calculate the detectable hazard ratio and corresponding sample sizes. A conservative index case recruitment period of 28 months was chosen after which a subsequent 6 months was allowed for follow up of index cases. Figure 4 shows the plot of detectable alternative hazard ratios of incidence density rate in the MDRTB arm relative to that in the control drug susceptible arm. Any combination of sample size, incidence density rate or detected hazard ratio in the top left or top right of the graph (Figure 26) will be statistically significant. The detectable alternative hazard ratio is plotted against experimental sample size (number of household contacts of MDRTB index patients) for the three incidence density rates in control drug susceptible tuberculosis houses (IC 0.025, IC 0.04 and IC 0.06) with a power of 0.8 and a two-tailed significance level of 0.05. Thus an incidence density rate of 0.04 in drug sensitive contacts with an MDRTB contact sample size of 800 would yield a statistically significant difference in incidence density rates between drug sensitive and MDRTB contacts only if the incidence of disease in MDRTB contacts was ≤0.6 or ≥2.0 times that among drug sensitive contacts.

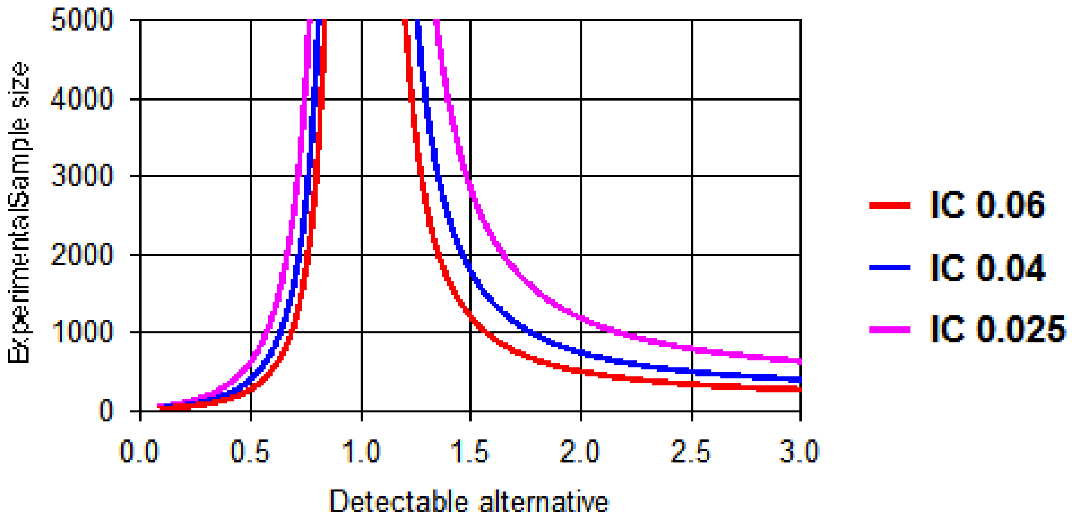

Figure 26 - A plot of detectable alternative hazard ratio.

The hazard ratio of incidence density rate in the experimental arm (MDRTB) relative to that in the control (drug susceptible) arm beyond which will be statistically significant, i.e. statistical significance is seen to the top left and top right of the graph. The detectable alternative hazard ratio is plotted against experimental sample size (number of household contacts in the MDRTB arm of the study) at three different hypothetical incidence density rates in the control arm of the study which were based on published evidence IC 0.025, 0.04 and 0.06.

The extent to which clustering (to account for correlated intra-household variance) reduced the power of the study could only be established by modelling simulations and was therefore not performed. It was accepted that clustering by household would slightly reduce the power of the study. Although not used in the pre-study power calculation, accounting for intra-household variance by clustering was used in the analysis.

7.2 Field Methods

7.2.1 Field Sites

A strong collaboration with the Peruvian Ministry of Health allowed the project to be set up in two of the four regions of metropolitan Lima. The first study site was located in the region of Callao to the North of Lima which has a population of ≈876,877 people, covers 146 square kilometres with a population density of 5608/km^2. The second study site was Lima South with a population of ≈1,455,946 million an area of 852 square kilometres and a population density of 1707/km^2. Both study sites have a tuberculosis incidence of approximately 100 new cases of tuberculosis per 100,000 inhabitants per year. In 2010 there were 150 new diagnoses of MDRTB in Lima South and 100 new diagnoses of MDRTB in Callao (Figure 2).

7.2.2 Definitions

MDRTB Index Case: A patient of any age with a diagnosis of pulmonary tuberculosis and microbiological confirmation of multidrug-resistance (MDR). MDR index cases are heterogeneous including both cases of new and retreatment tuberculosis. The questionnaire was designed to help delineate these groups so that the findings are more clearly defined.

Approximately 24% of previously treated cases in Peru have MDRTB, and MDRTB may evolve as a consequence of treatment failure in a previously drug susceptible patient. Hence it was expected that a significant proportion of MDRTB index cases would initially have had drug susceptible disease. It was impossible to determine the exact moment at which patients became drug resistant because of the lengthy time delay between serial sputum samples, therefore it was impossible to know the exact length of the infectious period of patients with MDRTB. For MDRTB the time from initial diagnosis of drug resistant tuberculosis was used as the start point for the survival analysis while the time of diagnosis of drug susceptible tuberculosis disease was used as the start point for drug susceptible index patients. Measuring exposure time from MDRTB diagnosis may

underestimate the infectious period (and therefore overestimate the incidence of disease) of MDRTB (as patients may have been living with MDRTB for months) whereas measuring exposure time from diagnosis of ‘any tuberculosis’ would over estimate the infectious period of MDRTB (and underestimate incidence).

Drug Susceptible Tuberculosis Index Case: A patient of any age with a diagnosis of pulmonary tuberculosis and a microbiological confirmation of tuberculosis susceptible to isoniazid and rifampicin. The drug susceptible index cases were selected to correspond temporally and geographically with the MDR index case and were also age matched.

Household Contacts: Anyone of any age who lived in the home of the index case for more than one day each week at the time of the index case tuberculosis diagnosis. If a contact that was living with the index case at the time of diagnosis subsequently left home, the health status, medical history and time of leaving was recorded in the questionnaire. If the contact died during follow-up, the date and cause of death was also recorded.

Second Case: A case of tuberculosis occurring among the household contacts after the diagnosis of tuberculosis in index case. The term "second case" was used for all incident cases (i.e. second, third or fourth household contacts with tuberculosis disease). This definition did not exclude the possibility of tuberculosis infection arising from outside the home or the reappearance of a previously latent tuberculosis infection. In order to reflect this uncertainty the term “second case” rather than “secondary case” was chosen to describe incident household cases of tuberculosis disease.

Tuberculosis disease in a household contact was defined as any household contact that started treatment for tuberculosis following the diagnosis of the index case. This was chosen in order not to exclude clinically diagnosed patients usually children who are often both smear and culture negative.

The Duration of Cough: The questionnaire recorded the duration of cough for the current disease episode.

7.2.3 Inclusion Criteria and Exclusion Criteria

Inclusion Criteria for Index Cases:

Pulmonary Tuberculosis

Culture confirmed MDR (or a confirmed drug susceptible control) Tuberculosis

Living in the study regions of Callao or Lima South.

New Diagnosis of Tuberculosis Disease

Exclusion Criteria for Index Cases:

Extra-pulmonary tuberculosis

Those without culture confirmation

Out of the study area

Imprisonment*

Patients living in an area too dangerous for the study team to enter†

Patients living on their own (with no household contacts)

Patients previously diagnosed as part of the same disease episode

*Ethical approval and institutional approval did not permit work in prisons. Approval would have needed to be sought through separate institutions outside the ministry of health.

†Only one health post in Callao (Centro de Salud Puerto Nuevo) had such high levels of gang violence and drug abuse that visiting the health centre was considered to be too great a risk to the safety of study staff.

7.2.4 Minimising Bias

Care was taken to avoid a bias in case detection: Ensuring that drug susceptible households were followed up identically (with the same frequency and at the same time) to drug resistant households minimized bias from preferential follow up of either group.

Bias as a consequence of a significant difference in health post attendance was also considered. The treatment course of MDRTB is 2 years and that of drug susceptible TB usually 6 months. Therefore despite the higher tendency for MDRTB patients to abandon therapy it is possible that they were more likely to be encountered at the health post than their drug susceptible counterparts. Following up one group at the health post and the other in their houses could lead to an ascertainment bias. The active follow up arm at the end of the study accounted for this by visiting all index patients in their homes.

Reporting bias was also minimised by the active follow up of all households. MDRTB index patients may have been less likely to report their un-well contacts because of fear of stigmatisation or concern that they have transmitted a more dangerous strain of disease.

Bias as a consequence of loss to follow up was possible and impossible to avoid. MDRTB patients are more likely to abandon treatment and more often have no fixed abode making underestimation of the incidence in MDRTB contacts a possibility because of this.

Demographic, environmental and clinical differences either among contacts or index patients will influence incidence among contacts and have not been regarded as biasing

the study. These factors will be recorded in the questionnaire and will help to account for any difference in incidence observed.

7.2.5 Ethical Approval and Institutional Approval

Both ethical approval from Universidad Peruana Cayetano Heredia and institutional approval from the Peruvian Ministry of Health were obtained before the study began. Ethical approval with identical translated documents was also obtained from the London School of Hygiene and Tropical medicine.

Consent forms specifically and explicitly asked permission from the patients participating in the study to store their sputum samples for up to 50 years. They also specified that the sputum samples would be used without the need to seek the consent of the patient for future related studies. This allowed for the creation of a well organised databank of MDRTB samples with open access for future tuberculosis drug resistance research. The ethics committee of UPCH requested that this aspect of the study was made as evident as possible to the participating patients and hence a tick box (Yes or No) with emboldened text was been inserted into the final approved consent form to ensure that this was the case.

7.2.6 Identifying Index MDRTB Patients and Drug Susceptible Controls

Drug resistant tuberculosis patients were diagnosed at the regional reference laboratories in both Callao and Lima South. New results were routinely uploaded onto 'netlab' (www.netlab.ins.gob.pe), the online laboratory database of the Peruvian National Institute of Health to which the study team had access. Each week, newly diagnosed patients who conformed to the entrance criteria were identified from the online database. Age matched, geographically matched contemporaneous drug susceptible controls were also selected at random from the available newly diagnosed drug susceptible cases identified on the online database.

The 'back-up' (decontaminated and centrifuged sputum sample) or the strain (normally plated on egg based Ogawa medium) was identified in the regional laboratory and

transported in a cooler to the research laboratory of Universidad Peruana Cayetano Heredia. Once the strain was confirmed to be growing in the laboratory, the cases were sought for interview.

The health post at which the index patient attended for directly observed therapy was identified in 'netlab' and a time to interview the index patient coordinated with the lead nurse of the tuberculosis program. The first interview and patient consent took place at the health post.

7.2.7 Questionnaire

The questionnaire (Appendix 5: Figure 38) was derived from previous studies of tuberculosis contacts, adapted following discussions with health staff of the Peruvian Tuberculosis program and field tested in a preliminary retrospective study of MDRTB household contacts [148]. The questionnaire was designed to reliably capture demographic, environmental, clinical and strain data of both the index case and the household contacts. Demographic variables include name, age, sex, work status, education, alcohol/tobacco/drugs use, history of incarceration, number of days spent in the house each week, relation to the index case, address, contact telephone and email address.

The socio-economic variables were based around the locally validated 'Necesidades Basicas Insatisfechas' scoring system used for the Peruvian National Census [341]. This simple 5 point scoring system acts as a proxy for the economic status of the index patient and his/her family. The five points consist of 1) The presence or absence of running water in the house 2) The presence or absence of a flushable toilet 3) Level of education 4) Crowding (people per room not including the bathroom or kitchen) and 5) The materials used for the construction of the floor, roof and walls.

The clinical data consisted of cough duration prior to diagnosis, a history of previous tuberculosis disease (defined as an episode of tuberculosis greater than 6 months prior to the onset of the present episode of tuberculosis disease) and treatment, history of

chemoprophylaxis, medical history, history of hospitalization, and side effects of therapy. A full treatment history was also recorded with dates of diagnosis, diagnostic method; culture, sputum smear or clinical, sputum smear result, treatment type (first line, standard empiric second line, selected empirical second line given to the contact based on the phenotype of the index case or individualised second line based on the phenotype of the patient).

Strain data included the date of collection of the first sputum sample sent for drug susceptibility testing and the three dates of the results of a possible 3 drug susceptibility tests and the corresponding results. The same data for incident cases among contacts was also recorded on the questionnaire.

7.2.8 Passive Follow-Up of Household Contacts

Index cases were followed up 6 monthly with an interview at the health post when they attended to take directly observed therapy. This frequency of follow-up allowed successful identification of incident cases with paired stored viable bacilli at the regional laboratory. If the patient completed therapy or did not attend the health post then the patient was interviewed in the house or by telephone. At the follow up interview, the index patients were asked about the wellbeing of household contacts, if any contacts had symptoms of tuberculosis (cough>2weeks, fever, night sweats or weight loss) they were be encouraged to attend the health post to provide a sputum sample and if they had already provided a sputum sample the results of the samples were sought and identified in the regional laboratory. Data about disease progression, changes in treatment scheme and sputum smear result were obtained from the treatment card.

7.2.9 Active Follow-Up of Household Contacts

To ensure that the incidence density rates were not underestimated by passive follow-up, all patients and their household contacts present were also interviewed at home. This took place 2 months after all index case recruitment stopped to allow time for symptomatic patients to be diagnosed by the local health post.

7.2.10 Concordant and Discordant Index/Contact Pairs

Pairs of index case and contact drug susceptibility tests were evaluated in order to assess the likely utility of an anti-tuberculous chemotherapeutic agent given to the household contact chosen on the basis of the index case phenotype ('tailored chemoprophylaxis'). Therefore if the susceptibility of the index case strain was matched by the susceptibility of the contact strain for every drug then the two strains were regarded as having perfectly concordant phenotypes. A different drug susceptibility result to any of the 11 drugs in a pair-wise comparison between the index case and the contact was regarded as being discordant. The number of discordant drugs between the index and contact were recorded.

7.2.11 HIV and Tuberculosis in Peru

All cases of tuberculosis were tested for HIV thus this data was available from the health record. The questionnaire also recorded data on HIV diagnosis in healthy contacts if it was known. Healthy contacts who were unaware of their diagnosis or who had not been tested were not tested for HIV. This decision was based on the low prevalence of HIV in Peru of less than 0.6% of the population and approximately 1.5% of Tuberculosis patients. It was accepted that this may have underestimated the prevalence of HIV among healthy contacts, however given the low prevalence of HIV disease it was felt that this omission was unlikely to bias the primary aims of the study.

It is often more difficult to establish a microbiological diagnosis in HIV patients [342] because of the tendency to have paucibacillary disease (as is also the case in children). Disregarding all cases of clinically diagnosed tuberculosis in contacts would have significantly underestimated the incidence of tuberculosis disease among contacts and was therefore not felt appropriate. Thus even undiagnosed HIV positive cases with clinically diagnosed tuberculosis were regarded as contributing to the incidence of disease. The proportion of clinically diagnosed contacts in each arm was thought not to be variable across the groups and indeed this was the case.

7.3 Statistical Analysis

Following double data entry into a Microsoft Access database and data cleaning, the results were analysed using Stata Statistical Software: Release 10. College Station, TX: StataCorp LP 2007. The follow-up time (used to calculate the incidence rate – number of disease episodes in contacts per person years of follow-up) began when the index case was diagnosed with tuberculosis (either MDRTB or drug susceptible TB). Household contacts who did not develop disease were censored (follow up time stopped) when the final household interview was undertaken, or when the contact left the house or was lost to follow-up. An event was defined as the diagnosis of tuberculosis disease in a household contact after the index case. Household clustered multivariate Cox regression was used to determine the significant predictors of a second case of tuberculosis disease in the household. Variables with a significance of $p \leq 0.2$ identified from univariate analysis were included in the multivariate analysis and any biologically plausible interactions were considered by determining the statistical significance of the product of the interacting terms in the model. The model was tested for violation of the proportional hazards assumption using Schoenfeld residual plots.

7.4 Concept and Study Design

The candidate conceived and designed this study. The previous work leading up to this study was jointly and equally conceived and designed by the candidate and his supervisor.

7.5 Study Staffing and Roles

Four nurses with previous experience of tuberculosis field work have been employed and trained by the candidate to interview participants and obtain consent. Until adequate numbers of index cases had been recruited to the study, the candidate worked alongside the nurses in the field. This work entails taking detailed histories from MDRTB patients and revising health records to capture data. To ensure maximum recruitment, study staff worked individually, two in Lima South, two in Callao and one in both field sites. The candidate also collaborated with the Ministry of Health, the local

health authorities and the regional laboratories to identify and deliver the strains to the laboratory in Cayetano Heredia.

Two staff with many years experience in a Mycobacterial research laboratory cultured the strains, extracted the DNA and genotyped the samples. The candidate supervised this work. The candidate was also in charge of all study logistics, project and staff management and statistical analysis. All these responsibilities were supervised by Professor David AJ Moore.

7.6 Study Aims

1) To measure the two-year incidence of secondary tuberculosis disease in household contacts of MDRTB patients; and compare it with the incidence in households with a drug susceptible tuberculosis index patient.

2) To quantify the proportion of index-second case household pairs with concordant drug susceptibility profiles and *Mycobacterium tuberculosis* strain fingerprints (phenotypic and genotypic matching).

3) To determine demographic, clinical and environmental predictors of concordance so that tailored preventive therapy can be targeted at subgroups of contacts most likely to share the same DST profile as the index case.
